# Genomic characterization of small cell carcinomas of the uterine cervix

**DOI:** 10.1002/1878-0261.12962

**Published:** 2021-05-13

**Authors:** Anne M. Schultheis, Ino de Bruijn, Pier Selenica, Gabriel S. Macedo, Edaise M. da Silva, Salvatore Piscuoglio, Achim A. Jungbluth, Kay J. Park, David S. Klimstra, Eva Wardelmann, Wolfgang Hartmann, Claus Dieter Gerharz, Mareike von Petersdorff, Reinhard Buettner, Jorge S. Reis‐Filho, Britta Weigelt

**Affiliations:** ^1^ Department of Pathology Memorial Sloan Kettering Cancer Center New York NY USA; ^2^ Department of Pathology University Hospital Cologne Germany; ^3^ Visceral Surgery Research Laboratory Clarunis Department of Biomedicine University of Basel Switzerland; ^4^ Department of Pathology University Hospital Muenster Germany; ^5^ Department of Pathology Bethesda Hospital Duisburg Germany; ^6^ Department of Pathology Marienkrankenhaus Hamburg Germany

**Keywords:** HPV, mutational signatures, neuroendocrine, small cell carcinoma, uterine cervix, whole‐exome sequencing

## Abstract

Small cell carcinoma (SCC) of the uterine cervix is a rare and aggressive form of neuroendocrine carcinoma, which resembles small cell lung cancer (SCLC) in its histology and poor survival rate. Here, we sought to define the genetic underpinning of SCCs of the uterine cervix and compare their mutational profiles with those of human papillomavirus (HPV)‐positive head and neck squamous cell carcinomas, HPV‐positive cervical carcinomas, and SCLCs using publicly available data. Using a combination of whole‐exome and targeted massively parallel sequencing, we found that the nine uterine cervix SCCs, which were HPV18‐positive (*n* = 8) or HPV16‐positive (*n* = 1), harbored a low mutation burden, few copy number alterations, and other than *TP53* in two cases no recurrently mutated genes. The majority of mutations were likely passenger missense mutations, and only few affected previously described cancer‐related genes. Using RNA‐sequencing, we identified putative viral integration sites on 18q12.3 and on 8p22 in two SCCs of the uterine cervix. The overall nonsilent mutation rate of uterine cervix SCCs was significantly lower than that of SCLCs, HPV‐driven cervical adeno‐ and squamous cell carcinomas, or HPV‐positive head and neck squamous cell carcinomas. Unlike SCLCs, which are reported to harbor almost universal *TP53* and *RB1* mutations and a dominant tobacco smoke‐related signature 4, uterine cervix SCCs rarely harbored mutations affecting these genes (2/9, 22% *TP53*; 0% *RB1*) and displayed a dominant aging (67%) or APOBEC mutational signature (17%), akin to HPV‐driven cancers, including cervical adeno‐ and squamous cell carcinomas and head and neck squamous cell carcinomas. Taken together, in contrast to SCLCs, which are characterized by highly recurrent *TP53* and *RB1* alterations, uterine cervix SCCs were positive for HPV leading to inactivation of the suppressors p53 and RB, suggesting that these SCCs are convergent phenotypes.

## Graphical abstract

Small cell carcinoma (SCC) of the uterine cervix is a rare and aggressive neuroendocrine carcinoma. Here, we show that uterine cervix SCCs are primarily HPV18‐positive. Using whole‐exome and/or targeted massively parallel sequencing, we found that uterine cervix SCCs have a low mutation burden, rarely harbor *TP53* and lack *RB1* mutations, display primarily dominant aging mutational signatures, and lack recurrent amplifications/homozygous deletions.

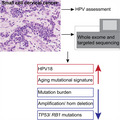

**Abbreviations**
HPV: human papillomavirus
SCC: small cell carcinoma
SCLC: small cell lung cancer
UCSCC: uterine cervix small cell carcinoma
WES: whole‐exome sequencing

## 1 Introduction

Cervical cancer is the second leading cause of cancer‐related deaths among women worldwide resulting in > 4000 deaths annually in the United States alone [1]. The most frequent histologic subtypes are squamous cell carcinomas and adenocarcinomas [2]. Although accounting for only 0.9% of invasive cervical carcinomas, uterine cervix small cell carcinomas (UCSCCs) are responsible for ~ 2.8% of the deaths in patients with cervical disease [3, 4].

The term small cell carcinoma (SCC) encompasses a set of highly aggressive neuroendocrine carcinomas, which preferentially not only affect the lung (95% of all SCCs) [5, 6] but may also be found in nearly any organ of the human body [7], in either pure or mixed forms. Irrespective of organ site, SCCs share distinct histologic features and clinical behavior. Histologically, SCCs are composed of small cells (up to three times the size of a resting lymphocyte) displaying little cytoplasm, oval‐ to spindle‐shaped nuclei with finely granular chromatin, and inconspicuous or absent nuclei and numerous mitotic figures. Areas of necrosis are frequently encountered [7]. For all SCCs, regardless of the anatomic site of origin, prognosis is poor. The reported median survival rates for patients with SCC range from 34 months in limited disease to 2 months for patients with extensive disease; most patients, however, present with advanced‐stage disease [8]. Given the biologic resemblance with small cell lung cancer (SCLC), treatment for patients with UCSCC is based on platinum‐containing regimens with optional radiation therapy [8, 9]. Akin to SCLC, despite good initial response rates, most patients with UCSCC relapse shortly after an initial response; hence, additional treatment options are urgently needed for these patients.

Pulmonary SCLCs have a highly characteristic molecular fingerprint, with almost universal *TP53‐* and *RB1‐*inactivating mutations [10], which are shared by SCCs of other anatomic sites such as the gastrointestinal (GI) tract and pancreas. Although UCSCCs are etiologically linked to infection with high‐risk human papillomaviruses (HPVs) [11, 12, 13], akin to other forms of cervical cancers, the molecular underpinning of UCSCCs remains to be fully elucidated. To date, loss of heterozygosity at specific gene/chromosomal regions using polymorphic microsatellite markers has revealed recurrent allelic imbalances affecting chromosomes 3q and 17p13, encompassing the *TP53* locus, but loss of heterozygosity of the *RB1* gene locus (13q14) was found to be rare [13, 14]. As for the repertoire of somatic mutations in UCSCCs, studies focusing on specific genes revealed recurrent *TP53* but no *KRAS* mutations [14], and targeted sequencing analyses found recurrent somatic mutations in *TP53*, *PIK3CA,* and *KRAS* [15, 16].

To date, it is unclear whether UCSCCs would have genetic features that recapitulate those of other common tumors of the uterine cervix (adenocarcinoma/ squamous cell carcinomas) or HPV‐driven cancers (e.g., head and neck carcinomas), or whether these tumors are more similar to SCCs of other anatomic sites such as SCLC. To address this question, we sought to define the constellation of somatic mutations, copy number alterations, and mutational signatures of UCSCCs using a combination of whole‐exome, targeted, and RNA‐sequencing. The mutational profiles of UCSCCs were compared with those from HPV‐positive head and neck squamous cell carcinomas and cervical carcinomas, and SCLCs.

## 2 Materials and methods

### 2.1 Case selection

The pathology files of University Hospital Cologne, Germany; University Hospital Muenster, Germany; Institute of Pathology Duisburg, Bethesda Hospital, Germany; and Memorial Sloan Kettering Cancer Center (MSK), New York, USA, were searched over a 20‐year period using the term ‘small cell’ and ‘neuroendocrine carcinoma’. Cases with origin in the lung were excluded after extensive chart review. For selected cases, data including tumor location, patient demographics, and information about prior treatment regimens were collected. In each case, the original hematoxylin‐and‐eosin (H&E)‐stained slide and immunohistochemistry (IHC) slides, if available, were reviewed. Cases lacking detailed clinical information or any doubt about being metastatic deposits from the lung were excluded. Cases were reviewed by four pathologists with an interest and expertise in neuroendocrine neoplasms (A.M.S., M.v.P., K.J.P., and D.S.K.). Diagnosis of SCC required the identification of tumors with cohesive round‐to‐ovoid cells, sparse cytoplasm, finely granulated nuclei, nuclear molding, and inconspicuous nucleoli. Cases were considered to be UCSCC if immunohistochemical expression of at least one neuroendocrine marker was detected and a high proliferation index (≥ 50%), assessed using Ki67, was observed. Sections from representative blocks of cases confirmed as UCSCCs were cut and H&E‐stained, and the block with the greatest tumor cell percentage was chosen for downstream analyses. A total number of nine UCSCCs were selected. All samples were anonymized prior to the analysis, and the approval by the institutional review boards (IRBs) of the respective contributing authors’ institutions was obtained. Informed consent was obtained from the patients following the requirements of the IRB‐approved protocols. This study is in compliance with the Declaration of Helsinki.

### 2.2 Immunohistochemistry

Representative 4‐μm‐thick formalin‐fixed paraffin‐embedded (FFPE) sections of each case were cut and subjected to ancillary immunohistochemical assessment using antibodies against cytokeratin (CK) AE1/E3, Ki67, chromogranin A and/or synaptophysin, and p16 (see Table S1 for details).

### 2.3 Nucleic acid extraction

For each of the nine cases included in this study, ten 8‐μm‐thick tumor and matched normal tissue sections (*n* = 2 frozen, *n* = 7 formalin‐fixed, paraffin‐embedded) were stained using nuclear fast red and subjected to microdissection by a pathologist (A.M.S.) using a sterile needle under a stereomicroscope (Olympus SZ61, Center Valley, PA, USA) to ensure a percentage of tumor cells greater than 80% and that the normal tissue was devoid of any tumor cells as previously described [17]. DNA was extracted using the DNeasy Blood and Tissue kit (Qiagen, Germantown, MD, USA), and RNA was extracted using TRIzol (Invitrogen, Thermo Fisher Scientific, Waltham, MA, USA), as previously described [18].

### 2.4 HPV detection

Type‐specific primers for the most commonly found HPV subtypes in cervical cancer (HPV16 and HPV18) were designed using Primer3. Standard PCR was performed using the AmpliTaq Gold 360 DNA Polymerase (Applied Biosystems, Thermo Fisher Scientific) following the manufacturers’ protocol. The forward and reverse primers (HPV16 Forward 5′‐GTACTGCAAGCAACAGTTACTGCGACGT, Reverse 5′‐CGACCGGTCCACCGACCCCT; HPV18 Forward 5′‐AACCTGTGTATATTGCAAGACAGTATTGGAACTTACA, Reverse 5′‐GATTCAACGGTTTCTGGCACCGC) were used for amplification of 312‐ and 251‐bp products, respectively, in the stable domain of the HPV. DNA samples from the HPV16‐positive CaSKi and HPV18‐positive HeLa cell lines were employed as positive controls [19].

### 2.5 Whole‐exome sequencing (WES) and targeted MSK‐IMPACT sequencing analysis

Microdissected tumor and matched normal DNA samples were subjected to WES (*n* = 6) or MSK‐IMPACT sequencing targeting 505 cancer‐related genes (*n* = 3) at MSK’s Integrated Genomics Operations (IGO), as previously described [20, 21, 22]. In brief, somatic single nucleotide variants (SNVs) were identified using MuTect [23], and small insertions and deletions (indels) were identified using a combination of Strelka, VarScan 2, Lancet, Platypus, and Scalpel [24, 25, 26, 27, 28], as previously described [20]. The potential functional effect of each SNV was investigated *in silico* using a combination of mutation prediction algorithms, as previously described [20, 21, 29]. copy number alterations and loss of heterozygosity were defined using FACETS [20, 21, 30], and the cancer cell fraction of each mutation (i.e., the bioinformatically inferred percentage of cancer cells harboring a given mutation) was inferred using ABSOLUTE (v1.0.6) [31]. Mutation hot spots were annotated according to Chang *et al*. [32]. Mutational signatures were defined for the six cases subjected to WES using deconstructSigs [33], as previously described [34].

### 2.6 Validation of mutations identified by WES

A subset of the somatic nonsynonymous mutations (*n* = 65) identified by WES was subjected to orthogonal validation using Sanger sequencing, as previously described [35] (Table S2). The validation rate was 62/65 (95.4%; Tables S3,S4). In addition, we subjected SCC1 and SCC4, initially analyzed by WES, to targeted MSK‐IMPACT sequencing (depth of sequencing: 595× SCC1T, 477× SCC4T); the somatic nonsynonymous mutations identified by WES sequencing affecting genes, which are also part of the MSK‐IMPACT sequencing panel, were all identified, and no additional somatic nonsynonymous mutations were detected (Fig. S1). These data support the robustness of our bioinformatics algorithms employed for the analysis of the samples included in this study.

### 2.7 Comparison with HPV‐positive head and neck, cervical, and SCLC

The frequencies of somatic mutations identified in UCSCCs studied here were compared with those of HPV‐positive head and neck squamous cell carcinomas (The Cancer Genome Atlas, TCGA, *n* = 29) [36], HPV‐positive cervical cancers (TCGA, *n* = 281) [37], and SCLC (*n* = 42 [38], *n* = 110 [10]). Publicly available whole‐exome sequencing (WES)‐derived mutational data were obtained from the NIH Genomic Data Commons (MC3 data; https://gdc.cancer.gov/about‐data/publications/pancanatlas) [39, 40] and/or the respective supplementary datasets. Frequencies of somatic mutations were compared using Fisher’s exact test; two‐tailed corrected *P* values < 0.05 were considered statistically significant. Statistical analyses were performed using r v3.1.2 and prism 7 (GraphPad Software, San Diego, CA, USA).

### 2.8 RNA‐sequencing

RNA of sufficient quality and quantity for RNA‐sequencing was obtained from the two frozen cases (SSC1 and SCC2); paired‐end massively parallel RNA‐sequencing was performed at MSK’s IGO using validated protocols, as previously described [18, 20]. The RNA‐sequencing data were used in three ways: First, TopHat (v2.0.13) [41] was employed for alignment, and deFuse (v0.6.1) [42] and ChimeraScan (v0.4.5) [43] for fusion detection. The oncogenic potential of each transcript was assessed with Oncofuse [44]. Second, viral integration sites were determined by aligning the RNA sequences against a combined reference of GRCh37 and the corresponding HPV as previously described [45]. A putative integration region is in between the pairs aligning to both genomes. Third, mutations identified by WES in cases SCC1 and SCC2 were validated in the RNA‐sequencing data using the SAMtools mpileup tool [46].

## 3 Results

### 3.1 Clinicopathologic features of UCSCCs

**Fig. 1:**
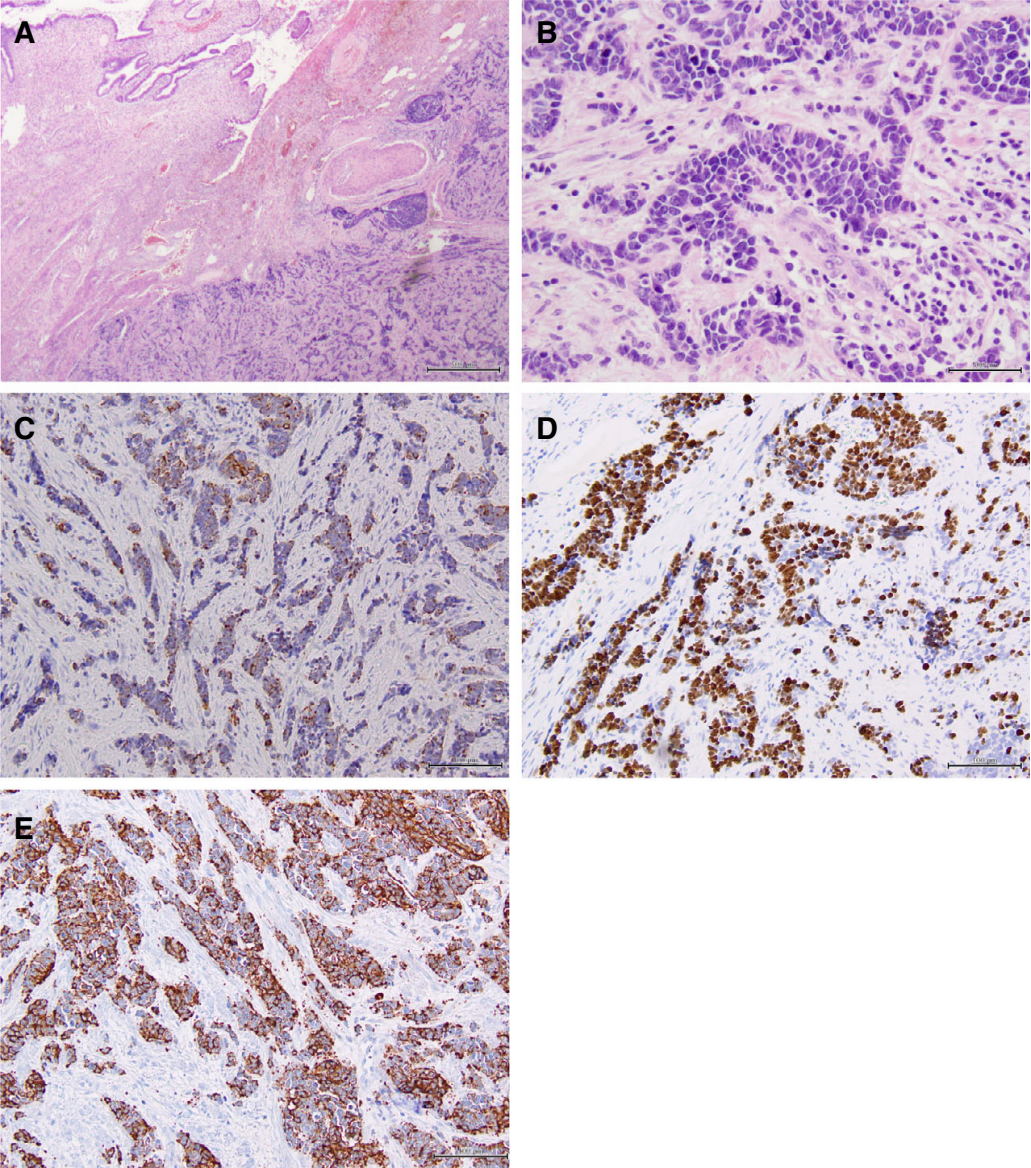
Histologic and immunohistochemical features of small cell carcinomas of the uterine cervix. Representative micrographs of a UCSCCs (case SCC6T). (A) Low‐power magnification of a H&E‐stained section showing the overall growth pattern of small cell carcinomas as dense tumor masses. (B) Higher‐power magnification of an H&E‐stained section highlighting the morphology of small cells, showing scant cytoplasm and small nuclei with finely granulated chromatin. Small cell carcinomas of the uterine cervix generally express (C) cytokeratin, (D) Ki67 and (E) chromogranin A. Scale bars (A, B) 500 µm and (C‐E) 100 µm.

The median age at diagnosis of the nine UCSCC patients was 40 years (range 27–55 years; Table 1). None of the patients had a history of prior malignancy, and all patients were investigated for the presence of cancers in other anatomic sites. All UCSCCs included in this study had the typical histologic features of SCCs in other anatomic locations, expressed at least one neuroendocrine marker and displayed numerous mitotic figures and high levels of proliferation as assessed by Ki67 immunohistochemistry (Fig. 1, Table 1). None had elements of adenocarcinoma or squamous cell carcinoma or a preinvasive neoplastic lesion. All cases were found to be positive for the presence of HPV, with eight UCSCCs being HPV18‐positive and one UCSCC being HPV16‐positive (Fig. S1), and expressed diffuse strong (block‐like) p16 (Table 1).

**Table 1:** Clinicopathologic information of small cell carcinomas of the uterine cervix included in this study. FF, flash‐frozen; NP, not performed.

ID	Ki67	Chromogranin A	Synaptophysin	CK AE1/3	p63	p16	HPV	Age at diagnosis (years)	Tissue	WES/ MSK‐IMPACT
SCC1T	70%	Negative	Positive	Dot‐like	Negative	Positive	HPV18	44	FF	WES^a^
SCC2T	85%	Positive	NP	Dot‐like	Negative	Positive	HPV18	28	FF	WES
SCC3T	50%	Positive	NP	Dot‐like	Negative	Positive	HPV18	49	FFPE	MSK‐IMPACT
SCC4T	50%	Positive	NP	Dot‐like	Negative	Positive	HPV18	35	FFPE	WES^a^
SCC5T	75%	Positive	Positive	Dot‐like	Negative	Positive	HPV18	55	FFPE	WES
SCC6T	80%	Positive	Positive	Dot‐like	Negative	Positive	HPV18	34	FFPE	WES
SCC7T	99%	Positive	NP	Focally dot‐like	Negative	Positive	HPV16	27	FFPE	WES
SCC8T	90%	Positive	Positive	Dot‐like	Negative	Positive	HPV18	43	FFPE	MSK‐IMPACT
SCC9T	70%	Positive	NP	Dot‐like	Negative	Positive	HPV18	40	FFPE	MSK‐IMPACT

^a^ For validation also subjected to targeted MSK‐IMPACT sequencing.

### 3.2 The repertoire of somatic genetic alterations in UCSCCs

**Fig. 2:**
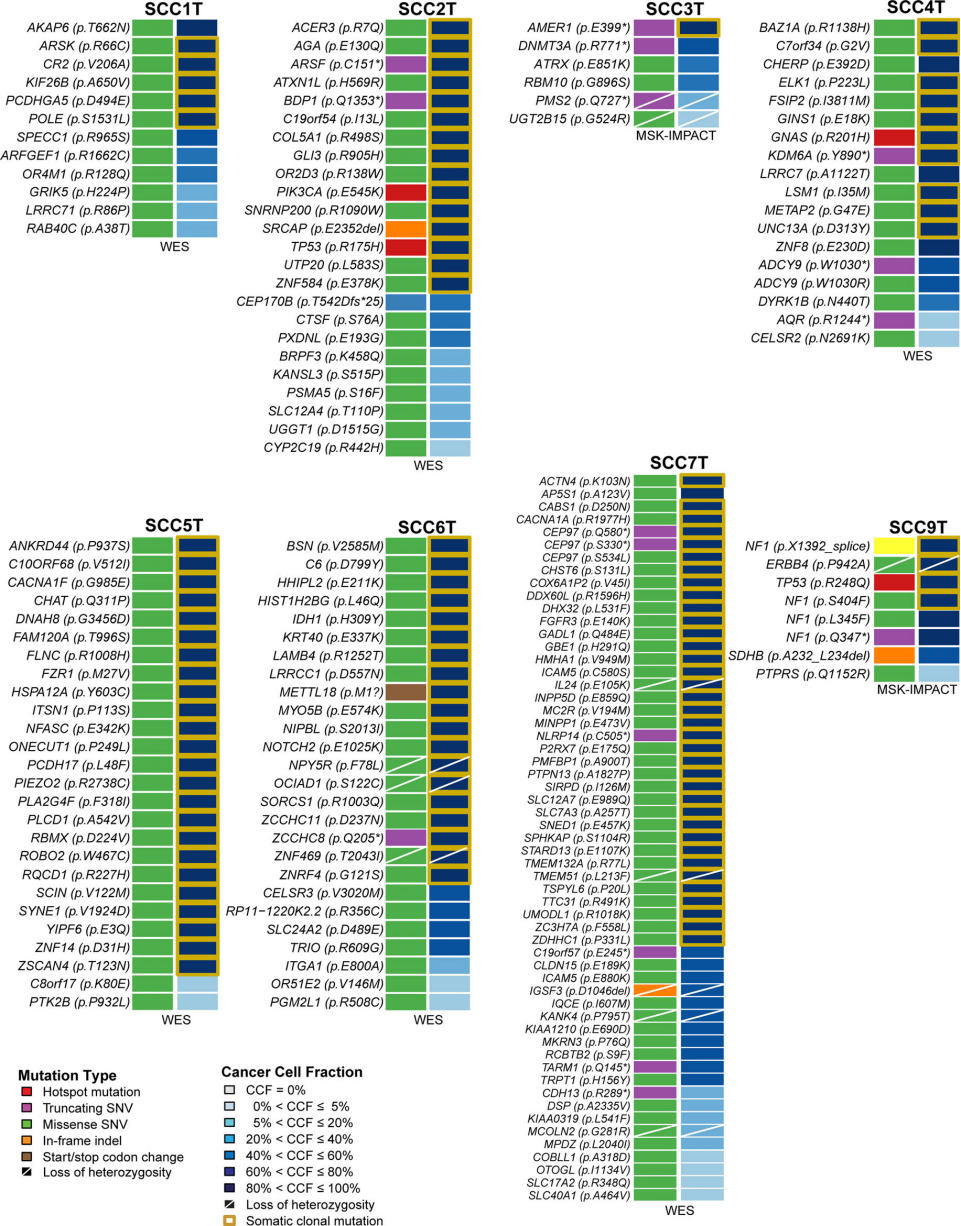
Somatic mutations identified and cancer cell fractions in small cell carcinomas of the uterine cervix using whole‐exome or targeted MSK‐IMPACT sequencing. Nonsynonymous somatic mutations (left) and cancer cell fractions of somatic mutations identified in the eight small cell carcinomas of the uterine cervix subjected to WES or MSK‐IMPACT sequencing targeting 505 cancer‐related genes. No nonsynonymous somatic mutations were identified in SCC8. Mutation type and cancer cell fractions (CCFs) are color‐coded according to the legend, with clonal mutations highlighted by an orange box. Loss of heterozygosity is depicted by a diagonal bar.

Six cases were subjected to WES and three cases to MSK‐IMPACT sequencing, with a median depth of WES sequencing coverage of 191× (range 139×–336×) for tumor and 146× (range 88×–181×) for normal samples, and of MSK‐IMPACT sequencing coverage of 405× (range 278×–408×) for tumor and 122× (range 115×–202×) for normal samples (Table S3). The UCSCCs subjected to WES harbored a median of 37.5 somatic mutations (range 21–84), of which a median of 25 (range 12–57) were nonsynonymous, and UCSCCs subjected to MSK‐IMPACT sequencing harbored a median of 10 somatic mutations (range 0–10), of which a median of 6 (range 0–8) were nonsynonymous (Table S3,S4). The repertoire of genetic alterations was heterogeneous in the nine UCSCCs studied. Two cases harbored *TP53* mutations (SCC2T and SCC9T); however, no other recurrent nonsynonymous somatic mutations were identified across the nine UCSCCs (Fig. 2, Table S4). SCC8T did not harbor any somatic mutations in the 505 cancer‐related genes tested. We found few mutations in previously described cancer‐related genes including *PIK3CA*, *TP53*, *NF1*, *IDH1*, *NOTCH2*, and *FGFR3* [39, 47, 48, 49]. The majority of mutations were likely passenger missense mutations, and few hot spot mutations were detected in the UCSCCs studied [32], including *TP53* R175H (SCC2T), *TP53* R248Q (SCC9T), *PIK3CA* E545K (SCC2T), and *GNAS* R844H (SCC4T), as well as some truncating mutations, including *PMS2* Q727* (SCC3T), *KDM6A* Y890* (SCC4T), and *NF1* Q357* (SCC9T; Fig. 2, Table S4). No *RB1* mutations were found.

**Fig. 3:**
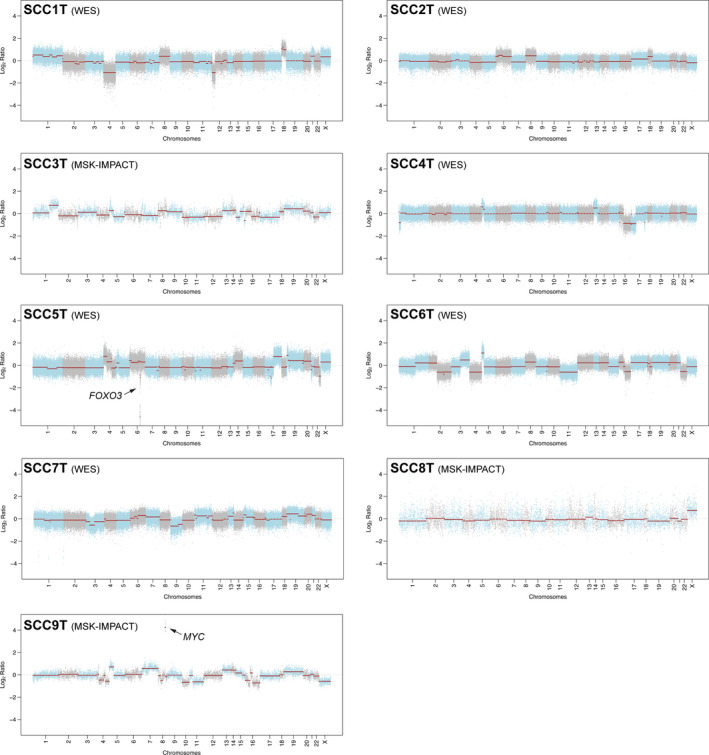
Copy number alterations in small cell carcinomas of the uterine cervix. Chromosome plots of the nine small cell carcinomas of the uterine cervix subjected to WES or MSK‐IMPACT sequencing targeting 505 cancer‐related genes. copy number log_2_ ratios are depicted on the *y*‐axis with the chromosome location on the *x*‐axis. Black arrows, amplification and homozygous deletion identified.

Copy number analysis revealed that akin to other virus‐induced cancers [50], UCSCCs displayed few gene copy number alterations. We only detected one *MYC* amplification in SCC9T, and SSC5T harbored a *FOXO3* homozygous deletion (Fig. 3). Analysis of the cancer cell fraction of the nonsynonymous somatic mutations identified in the eight UCSCCs harboring mutations (SCC8T had no mutations) using ABSOLUTE [31] revealed intratumor genetic heterogeneity, with a median of 41% (range 8–83%) of mutations in a given case being subclonal (Fig. 2, Table S4).

We obtained RNA of sufficient quality and quantity for RNA‐sequencing for two UCSCCs (SSC1 and SSC2), which did not reveal predicted in‐frame fusion transcripts/ read‐throughs with high driver probability (Oncofuse, > 0.9) and with adequate encompassing and spanning reads (> 5) (Table S5). We did, however, identify putative viral integration sites, which are paired reads that have one mate aligning to the human reference and the other mate to HPV18. We identified such sites for SCC1 on 18q12.3 at the non‐annotated positions of 39411292, 39497187, and 39401088 with the mate aligning to HPV18 at positions 882 (E7), 2280 (E1), and 3669 (E2), respectively. For SCC2, the viral integration sites were located on 8p22 in RP11‐89M16.1‐002, a lncRNA, at the positions 129517175, 129518183, 129517009, and 129509523 with the mate in HPV18 at 2603 (E1), 4908 (L2), 5996 (L1), and 5998 (L1), respectively (Table S6).

### 3.3 UCSCCs display somatic genetic alterations distinct from those of SCLCs

**Fig. 4:**
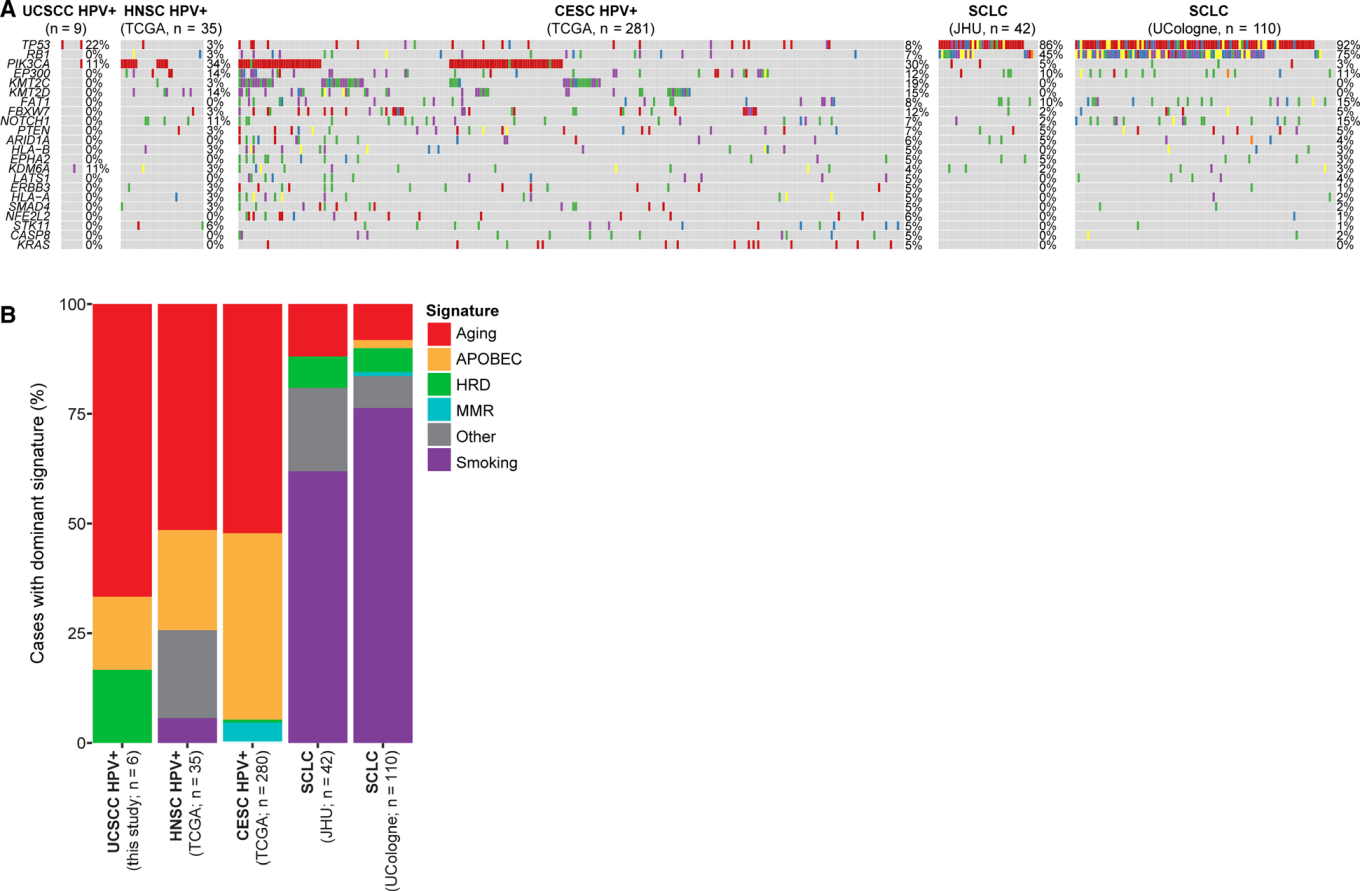
Comparison of the mutational profiles and mutational signatures of small cell carcinomas of the uterine cervix with SCLCs, HPV‐positive head and neck carcinomas, and HPV‐positive cervical carcinomas. (A) Comparison of somatic nonsynonymous mutations in small cell carcinomas of the uterine cervix (this study) with those of SCLCs and HPV‐driven head and neck and HPV‐driven cervical cancers (adenocarcinoma/ squamous cell carcinomas), showing the top 15 recurrently mutated genes across all studies. (B) Mutational signatures in small cell carcinomas of the uterine cervix subjected to WES (*n* = 6; this study), SCLCs, and HPV‐driven head and neck and HPV‐driven cervical cancers (adenocarcinoma/ squamous cell carcinomas). HNSC, head and neck squamous cell cancer; CESC, cervical adenocarcinoma/ squamous cell carcinomas.

The overall mutation rate of UCSCCs subjected to WES was 0.72 mutations/Mb with an average rate for nonsilent mutations of 0.49/Mb, which is significantly lower (*P* < 0.001, Benjamini–Hochberg test) than the mutation rates described for SCLCs (overall mutation rate 7.37/Mb), HPV‐driven cervical adeno‐ and squamous cell carcinomas (nonsilent mutation rate 3.7/Mb), or HPV‐positive head and neck cancers (overall mutation rate 2.28/Mb) [38, 51, 52, 53]. *TP53* mutations are present in the vast majority of SCLCs (up to 98%) [10, 38, 40], whereas HPV‐driven cancers, including previously reported cervical adeno‐ and squamous cell carcinomas [37], head and neck squamous cell carcinomas [36], and the UCSCCs from this study, displayed lower *TP53* mutation frequencies (3–22%; Fig. 4A). In addition, *RB1* somatic mutations have been reported to be frequent in the SCLCs (up to 98%) [10, 38, 40] but were found to be rare in the HPV‐positive UCSCCs analyzed here (0%), and in previously reported HPV‐positive cervical adeno‐ and squamous cell carcinomas (7%) or HPV‐positive head and neck squamous cell carcinomas (3%) [36, 37] (Fig. 4A). Conversely, a higher frequency of *PIK3CA* mutations occurred in previously reported HPV‐positive cervical adeno‐ and squamous cell carcinomas, head and neck cancers [36, 37], and the UCSCC analyzed here (17–34%) as compared to previously described SCLCs (3–5%; Fig. 4A). Mutations in the chromatin remodeling genes *KMT2C* and *KMT2D* were reported in cervical adeno‐ and squamous cell carcinomas (19% and 15%, respectively), but were absent in previously reported SCLCs and in the UCSCCs analyzed here (Fig. 4A).

We next defined the mutational signatures of the UCSCCs subjected to WES using all somatic SNVs identified. Four of the cases studied here displayed a dominant aging signature (i.e., signature 1), while SCC7, the only case that tested positive for HPV16, displayed an APOBEC signature (i.e., signature 2; Fig. 4B) [54]. APOBEC3A has been suggested to possess antiviral effects by inhibition of HPV E6 and E7 expression through cytidine deaminase [55]. Consistent with the repertoire of somatic mutations, we observed that like UCSCCs, HPV‐positive cervical adeno‐ and squamous cell carcinomas and HPV‐positive head and neck cancers preferentially displayed a dominant mutational signature 1 associated with aging and the APOBEC‐related signatures 2 and 13. In contrast, the majority of SCLCs displayed a dominant signature 4 associated with tobacco smoke, and only a few cases harbored a dominant aging‐related mutational signature (Fig. 4B).

## 4 Discussion

Massively parallel sequencing studies have focused on the characterization of the genetic landscape of many tumors of different organ sites, which revealed that cancers from different organs sites often share genetic features, whereas, conversely, different cancer types from the same organ can be quite distinct at the genetic level [39, 56]. For example, *TP53* mutations and high levels of gene copy number alterations have been found in high‐grade serous ovarian, serous endometrial, and basal‐like breast carcinomas [48, 57, 58]. Conversely, there are alterations in genes, whose effects may differ depending on the organ site/ cell of origin. A prime example is provided by the *NOTCH* gene family, which is inactivated in some squamous cell cancers of the lung, head and neck [53], skin [59], and cervix [60] but is activated by mutation in leukemias [61].

Small cell neuroendocrine carcinomas, irrespective of their site of origin, share the same histologic features. Other than recurrent *TP53* and *RB1* mutations in small cell neuroendocrine carcinomas of the GI tract and pancreas [62, 63], little is known about the genetic commonalities or differences in these tumors originating outside of the lung. Due to morphologic and biologic similarities between SCCs of the uterine cervix and SCLCs, it has long been hypothesized that these tumors might be genetically similar and that they may share affected genes and/or pathways [7]. Another hypothesis that has been brought forward is that adenocarcinomas and/or squamous cell carcinomas may progress to SCCs [64], suggesting that they would harbor a similar mutational repertoire plus additional mutations characteristic for SCCs.

Here, we characterized nine UCSCCs at the histologic and the molecular level to assess whether these tumors are genetically related to their morphologic and biologic counterparts in the lung, other common types of cervical cancer, or whether these tumors are genetically more similar to other HPV‐driven carcinomas, such as carcinomas of the head and neck. Our analyses demonstrated that UCSCCs harbor a low overall mutation burden, few copy number alterations, and no highly recurrently mutated genes. Only 2 of 9 UCSCCs studied here harbored *TP53* mutations and 1/9 a *PIK3CA* mutation, whereas other targeted sequencing studies reported 4 of 10 and 5 of 44 UCSCCs to harbor *TP53* mutations, and 3 of 10 and 8 of 44 to have *PIK3CA* mutations [15, 16]. Another study on five UCSCCs found alterations in the AKT/mTOR pathway, including *PTEN* and *TSC1/2* mutations [65]. Further studies are warranted to capture the entire genetic complexity/ heterogeneity of UCSCCs. Virus‐negative tumors require several genetic events to induce malignant transformation, whereas the viral integration is a strong oncogenic event in virus‐positive tumors. Notably, we found that UCSCCs harbor even fewer genetic alterations than HPV‐positive head and neck cancers or HPV‐positive adeno‐ and squamous cell carcinomas of the uterine cervix. We further observed that like other HPV‐driven tumors, however, UCSCCs lacked *RB1* mutations and had a low frequency of *TP53* mutations (2/9) and displayed mutational signatures associated with aging or the activity of the APOBEC family of deaminases, whereas SCLCs generally displayed tobacco smoke‐related mutational signatures.

All but one UCSCC studied here were HPV18‐positive, in contrast to common‐type cervical cancers, which most commonly are HPV16‐positive [37, 66]. High‐risk HPVs are double‐stranded DNA viruses that infect epithelial cells [11, 67]. Tumorigenesis by high‐risk HPVs is driven by their two main viral oncogenes, E6 and E7, which inactivate p53 and pRb, respectively, leading to cell‐cycle deregulation and inhibition of p53‐mediated apoptosis [11, 67]. E7 binds pRb, targeting it toward proteasomal degradation, in turn releasing the E2F transcription factor, resulting in CDKN2A (or p16) overexpression and cell‐cycle progression [11, 67]. SCLCs harbor recurrent genetic alterations affecting the p53 and the pRB pathways [10], with biallelic inactivation of *TP53* and *RB1* being found in the vast majority of cases. Given that the UCSCCs analyzed here harbored HPV18 or HPV16 and that the viral oncogenes E6 and E7 inactivate p53 and pRB, we anticipated that these tumors would display wild‐type *TP53* and *RB1* but would harbor functional loss of these tumor suppressor genes. Consistent with this notion, only SCC2T and SCC9T harbored *TP53* hotspot mutations. Hence, despite the lack of *TP53* and *RB1* somatic mutations in the UCSCCs analyzed here, they likely display inactivation of the protein products of these genes akin to SCLCs.

This study has several limitations. UCSCCs are aggressive tumors, which are only rarely resected, and the availability of clinical samples for research is therefore limited; hence, the sample size of the current study is small. The genetic analysis of nine cases performed here, however, provided us with novel insights on their landscape of mutations, gene copy number alterations, and mutational signatures. In addition, we only performed whole‐exome and targeted sequencing analyses of the UCSCCs and RNA‐sequencing for two cases; we therefore cannot rule out that noncoding alterations and/or changes at the epigenetic level may play a role in UCSCCs.

## 5 Conclusions

In summary, we demonstrate here that UCSCCs are characterized by few nonrecurrent mutations and few copy number alterations, and display aging and APOBEC‐related mutational signatures, akin to other forms of HPV‐related malignancies. In contrast to SCLCs, which are characterized by *TP53* and *RB1* alterations, UCSCCs were found to be positive for the presence of HPV, which targets and inactivates the suppressors p53 and RB.

## Conflict of interest

DSK is a founder, consultant, and equity holder of Paige. AI and receives royalties from UpToDate and the American Registry of Pathology, outside of this work. JSR‐F reports receiving personal/consultancy fees from Goldman Sachs, Repare Therapeutics, and Paige.AI, membership of the scientific advisory boards of VolitionRx, Repare Therapeutics, and Paige.AI, membership of the Board of Directors of Grupo Oncoclinicas, and ad hoc membership of the scientific advisory boards of Roche Tissue Diagnostics, Ventana Medical Systems, Novartis, Genentech, and InVicro, outside the scope of this study. BW reports ad hoc membership of the scientific advisory board of Repare Therapeutics, outside the scope of the submitted work. The remaining authors have no conflicts of interest to declare.

## Author contributions

AMS, BW, and JSR‐F conceived and supervised the study. EW, WH, CDG, BW, and RB provided samples. AMS, MvP, DSK, and KJP performed histologic review. AMS performed sample microdissection. AMS, GSM, EMdS, SP, and BW carried out experiments. AAJ performed immunohistochemical analyses. IdB and PS performed bioinformatics analyses. AMS, IdB, PS, BW, and JSR‐F interpreted results and drafted the manuscript. All authors reviewed and approved the final version of the manuscript.

## Supporting information


**Fig. S1**. Comparison of whole‐exome and high‐depth targeted sequencing of uterine cervix small cell carcinomas SCC1 and SCC4.
**Fig. S2**. Detection of HPV16 and HPV18 DNA in uterine cervix small cell carcinomas.
**Table S1**. Antibody clones, dilutions, antigen retrievals and scoring used for the immunohistochemical analyses performed.
**Table S2**. Primers for Sanger sequencing validation of mutations identified by whole‐exome sequencing.
**Table S3**. Sequencing statistics, number of somatic mutations identified and validation rates.
**Table S4**. Non‐synonymous somatic mutations identified in small cell carcinomas of the uterine cervix using whole‐exome and MSK‐IMPACT targeted sequencing.
**Table S5**. Putative in‐frame fusion transcripts with driver probability > 0.1 identified in SCC1 and SCC2 using RNA‐sequencing.
**Table S6**. Putative HPV integration sites based on RNA‐sequencing.

## Data Availability

The data that support the findings of this study are available in Tables S3–S6 of this article, and were derived from the following resources in the public domain: TCGA WES‐based MC3 data of HPV‐positive head and neck and HPV‐positive common‐type cervical cancers were obtained from the NIH Genomic Data Commons Portal (https://gdc.cancer.gov/about‐data/publications/pancanatlas) [36, 37, 39]. WES‐based SCLC data were obtained from Tables S3,S4 from Rudin *et al*. [38] and Table S3 from George *et al*. [10].

## References

[1] Siegel R , Ma J , Zou Z & Jemal A (2014) Cancer statistics, 2014. CA Cancer J Clin 64, 9–29. 24399786 10.3322/caac.21208

[2] Wang SS , Sherman ME , Hildesheim A , Lacey JV Jr & Devesa S (2004) Cervical adenocarcinoma and squamous cell carcinoma incidence trends among white women and black women in the United States for 1976–2000. Cancer 100, 1035–1044. 14983500 10.1002/cncr.20064

[3] Chen J , Macdonald OK & Gaffney DK (2008) Incidence, mortality, and prognostic factors of small cell carcinoma of the cervix. Obstet Gynecol 111, 1394–1402. 18515524 10.1097/AOG.0b013e318173570b

[4] Atienza‐Amores M , Guerini‐Rocco E , Soslow RA , Park KJ & Weigelt B (2014) Small cell carcinoma of the gynecologic tract: a multifaceted spectrum of lesions. Gynecol Oncol 134, 410–418. 24875120 10.1016/j.ygyno.2014.05.017

[5] van Meerbeeck JP , Fennell DA & De Ruysscher DK (2011) Small‐cell lung cancer. Lancet 378, 1741–1755. 21565397 10.1016/S0140-6736(11)60165-7

[6] Richardson RL & Weiland LH (1982) Undifferentiated small cell carcinomas in extrapulmonary sites. Semin Oncol 9, 484–496. 6302908

[7] Frazier SR , Kaplan PA & Loy TS (2007) The pathology of extrapulmonary small cell carcinoma. Semin Oncol 34, 30–38. 17270663 10.1053/j.seminoncol.2006.11.017

[8] Brennan SM , Gregory DL , Stillie A , Herschtal A , Mac Manus M & Ball DL (2010) Should extrapulmonary small cell cancer be managed like small cell lung cancer? Cancer 116, 888–895. 20052730 10.1002/cncr.24858

[9] Walenkamp AM , Sonke GS & Sleijfer DT (2009) Clinical and therapeutic aspects of extrapulmonary small cell carcinoma. Cancer Treat Rev 35, 228–236. 19068273 10.1016/j.ctrv.2008.10.007

[10] George J , Lim JS , Jang SJ , Cun Y , Ozretic L , Kong G , Leenders F , Lu X , Fernandez‐Cuesta L , Bosco G *et al*. (2015) Comprehensive genomic profiles of small cell lung cancer. Nature 524, 47–53. 26168399 10.1038/nature14664 PMC4861069

[11] zur Hausen H (2009) Papillomaviruses in the causation of human cancers ‐ a brief historical account. Virology 384, 260–265. 19135222 10.1016/j.virol.2008.11.046

[12] Kashiwabara K & Nakajima T (1992) Detection of human papillomavirus DNA in invasive cervical cancers by the polymerase chain reaction and its clinical significance. Acta Pathol Jpn 42, 876–883. 1337817 10.1111/j.1440-1827.1992.tb01893.x

[13] Mannion C , Park WS , Man YG , Zhuang Z , Albores‐Saavedra J & Tavassoli FA (1998) Endocrine tumors of the cervix: morphologic assessment, expression of human papillomavirus, and evaluation for loss of heterozygosity on 1p,3p, 11q, and 17p. Cancer 83, 1391–1400. 9762941 10.1002/(sici)1097-0142(19981001)83:7<1391::aid-cncr17>3.0.co;2-#

[14] Wistuba II , Thomas B , Behrens C , Onuki N , Lindberg G , Albores‐Saavedra J & Gazdar AF (1999) Molecular abnormalities associated with endocrine tumors of the uterine cervix. Gynecol Oncol 72, 3–9. 9889022 10.1006/gyno.1998.5248

[15] Xing D , Zheng G , Schoolmeester JK , Li Z , Pallavajjala A , Haley L , Conner MG , Vang R , Hung CF , Wu TC *et al*. (2018) Next‐generation sequencing reveals recurrent somatic mutations in small cell neuroendocrine carcinoma of the uterine cervix. Am J Surg Pathol 42, 750–760. 29505425 10.1097/PAS.0000000000001042 PMC5943084

[16] Frumovitz M , Burzawa JK , Byers LA , Lyons YA , Ramalingam P , Coleman RL & Brown J (2016) Sequencing of mutational hotspots in cancer‐related genes in small cell neuroendocrine cervical cancer. Gynecol Oncol 141, 588–591. 27079212 10.1016/j.ygyno.2016.04.001 PMC4877250

[17] Martelotto LG , De Filippo MR , Ng CK , Natrajan R , Fuhrmann L , Cyrta J , Piscuoglio S , Wen HC , Lim RS , Shen R *et al*. (2015) Genomic landscape of adenoid cystic carcinoma of the breast. J Pathol 237, 179–189. 26095796 10.1002/path.4573 PMC4676955

[18] Piscuoglio S , Burke KA , Ng CK , Papanastasiou AD , Geyer FC , Macedo GS , Martelotto LG , de Bruijn I , De Filippo MR , Schultheis AM *et al*. (2016) Uterine adenosarcomas are mesenchymal neoplasms. J Pathol 238, 381–388. 26592504 10.1002/path.4675 PMC4986517

[19] Meissner JD (1999) Nucleotide sequences and further characterization of human papillomavirus DNA present in the CaSki, SiHa and HeLa cervical carcinoma cell lines. J Gen Virol 80 (Pt 7), 1725–1733. 10423141 10.1099/0022-1317-80-7-1725

[20] Pareja F , Lee JY , Brown DN , Piscuoglio S , Gularte‐Merida R , Selenica P , Da Cruz PA , Arunachalam S , Kumar R , Geyer FC *et al*. (2019) The genomic landscape of mucinous breast cancer. J Natl Cancer Inst 111, 737–741. 30649385 10.1093/jnci/djy216 PMC6624163

[21] Moukarzel LA , Da Cruz Paula A , Ferrando L , Hoang T , Sebastiao APM , Pareja F , Park KJ , Jungbluth AA , Capella G , Pineda M *et al*. (2020) Clonal relationship and directionality of progression of synchronous endometrial and ovarian carcinomas in patients with DNA mismatch repair‐deficiency associated syndromes. Mod Pathol. 10.1038/s41379-020-00721-6. Online ahead of print. PMC8076061 33328602

[22] Cheng DT , Mitchell TN , Zehir A , Shah RH , Benayed R , Syed A , Chandramohan R , Liu ZY , Won HH , Scott SN *et al*. (2015) Memorial sloan Kettering‐integrated mutation profiling of actionable cancer targets (MSK‐IMPACT): a hybridization capture‐based next‐generation sequencing clinical assay for solid tumor molecular oncology. J Mol Diagn 17, 251–264. 25801821 10.1016/j.jmoldx.2014.12.006 PMC5808190

[23] Cibulskis K , Lawrence MS , Carter SL , Sivachenko A , Jaffe D , Sougnez C , Gabriel S , Meyerson M , Lander ES & Getz G (2013) Sensitive detection of somatic point mutations in impure and heterogeneous cancer samples. Nat Biotechnol 31, 213–219. 23396013 10.1038/nbt.2514 PMC3833702

[24] Koboldt DC , Zhang Q , Larson DE , Shen D , McLellan MD , Lin L , Miller CA , Mardis ER , Ding L & Wilson RK (2012) VarScan 2: somatic mutation and copy number alteration discovery in cancer by exome sequencing. Genome Res 22, 568–576. 22300766 10.1101/gr.129684.111 PMC3290792

[25] Saunders CT , Wong WS , Swamy S , Becq J , Murray LJ & Cheetham RK (2012) Strelka: accurate somatic small‐variant calling from sequenced tumor‐normal sample pairs. Bioinformatics 28, 1811–1817. 22581179 10.1093/bioinformatics/bts271

[26] Narzisi G , Corvelo A , Arora K , Bergmann EA , Shah M , Musunuri R , Emde A‐K , Robine N , Vacic V & Zody MC (2018) Genome‐wide somatic variant calling using localized colored de Bruijn graphs. Comms Bio 1, 1–9. 10.1038/s42003-018-0023-9 PMC6123722 30271907

[27] Rimmer A , Phan H , Mathieson I , Iqbal Z , Twigg SRF , WGS500 Consortium , Wilkie AOM , McVean G & Lunter G (2014) Integrating mapping‐, assembly‐ and haplotype‐based approaches for calling variants in clinical sequencing applications. Nat Genet 46, 912–918. 25017105 10.1038/ng.3036 PMC4753679

[28] Fang H , Bergmann EA , Arora K , Vacic V , Zody MC , Iossifov I , O'Rawe JA , Wu Y , Jimenez Barron LT , Rosenbaum J *et al*. (2016) Indel variant analysis of short‐read sequencing data with Scalpel. Nat Protoc 11, 2529–2548. 27854363 10.1038/nprot.2016.150 PMC5507611

[29] Martelotto LG , Ng C , De Filippo MR , Zhang Y , Piscuoglio S , Lim R , Shen R , Norton L , Reis‐Filho JS & Weigelt B (2014) Benchmarking mutation effect prediction algorithms using functionally validated cancer‐related missense mutations. Genome Biol 15, 484. 25348012 10.1186/s13059-014-0484-1 PMC4232638

[30] Shen R & Seshan VE (2016) FACETS: allele‐specific copy number and clonal heterogeneity analysis tool for high‐throughput DNA sequencing. Nucleic Acids Res 44, e131. 27270079 10.1093/nar/gkw520 PMC5027494

[31] Carter SL , Cibulskis K , Helman E , McKenna A , Shen H , Zack T , Laird PW , Onofrio RC , Winckler W , Weir BA *et al*. (2012) Absolute quantification of somatic DNA alterations in human cancer. Nat Biotechnol 30, 413–421. 22544022 10.1038/nbt.2203 PMC4383288

[32] Chang MT , Bhattarai TS , Schram AM , Bielski CM , Donoghue MTA , Jonsson P , Chakravarty D , Phillips S , Kandoth C , Penson A *et al*. (2018) Accelerating discovery of functional mutant alleles in cancer. Cancer Discov 8, 174–183. 29247016 10.1158/2159-8290.CD-17-0321 PMC5809279

[33] Rosenthal R , McGranahan N , Herrero J , Taylor BS & Swanton C (2016) DeconstructSigs: delineating mutational processes in single tumors distinguishes DNA repair deficiencies and patterns of carcinoma evolution. Genome Biol 17, 31. 26899170 10.1186/s13059-016-0893-4 PMC4762164

[34] Ashley CW , Da Cruz PA , Kumar R , Mandelker D , Pei X , Riaz N , Reis‐Filho JS & Weigelt B (2019) Analysis of mutational signatures in primary and metastatic endometrial cancer reveals distinct patterns of DNA repair defects and shifts during tumor progression. Gynecol Oncol 152, 11–19. 30415991 10.1016/j.ygyno.2018.10.032 PMC6726428

[35] Weinreb I , Piscuoglio S , Martelotto LG , Waggott D , Ng CK , Perez‐Ordonez B , Harding NJ , Alfaro J , Chu KC , Viale A *et al*. (2014) Hotspot activating PRKD1 somatic mutations in polymorphous low‐grade adenocarcinomas of the salivary glands. Nat Genet 46, 1166–1169. 25240283 10.1038/ng.3096 PMC10208689

[36] Cancer Genome Atlas Network (2015) Comprehensive genomic characterization of head and neck squamous cell carcinomas. Nature 517, 576–582. 25631445 10.1038/nature14129 PMC4311405

[37] Cancer Genome Atlas Research Network (2017) Integrated genomic and molecular characterization of cervical cancer. Nature 543, 378–384. 28112728 10.1038/nature21386 PMC5354998

[38] Rudin CM , Durinck S , Stawiski EW , Poirier JT , Modrusan Z , Shames DS , Bergbower EA , Guan Y , Shin J , Guillory J *et al*. (2012) Comprehensive genomic analysis identifies SOX2 as a frequently amplified gene in small‐cell lung cancer. Nat Genet 44, 1111–1116. 22941189 10.1038/ng.2405 PMC3557461

[39] Bailey MH , Tokheim C , Porta‐Pardo E , Sengupta S , Bertrand D , Weerasinghe A , Colaprico A , Wendl MC , Kim J , Reardon B *et al*. (2018) Comprehensive characterization of cancer driver genes and mutations. Cell 173, 371–385.e318. 29625053 10.1016/j.cell.2018.02.060 PMC6029450

[40] Gao J , Aksoy BA , Dogrusoz U , Dresdner G , Gross B , Sumer SO , Sun Y , Jacobsen A , Sinha R , Larsson E *et al*. (2013) Integrative analysis of complex cancer genomics and clinical profiles using the cBioPortal. Sci Signal 6, pl1. 23550210 10.1126/scisignal.2004088 PMC4160307

[41] Trapnell C , Pachter L & Salzberg SL (2009) TopHat: discovering splice junctions with RNA‐Seq. Bioinformatics 25, 1105–1111. 19289445 10.1093/bioinformatics/btp120 PMC2672628

[42] McPherson A , Hormozdiari F , Zayed A , Giuliany R , Ha G , Sun MGF , Griffith M , Heravi Moussavi A , Senz J , Melnyk N *et al*. (2011) deFuse: an algorithm for gene fusion discovery in tumor RNA‐seq data. PLoS Comput Biol 7, e1001138. 21625565 10.1371/journal.pcbi.1001138 PMC3098195

[43] Iyer MK , Chinnaiyan AM & Maher CA (2011) ChimeraScan: a tool for identifying chimeric transcription in sequencing data. Bioinformatics 27, 2903–2904. 21840877 10.1093/bioinformatics/btr467 PMC3187648

[44] Shugay M , Ortiz de Mendibil I , Vizmanos JL & Novo FJ (2013) Oncofuse: a computational framework for the prediction of the oncogenic potential of gene fusions. Bioinformatics 29, 2539–2546. 23956304 10.1093/bioinformatics/btt445

[45] Wang Q , Jia P , Zhao Z & Parkin DM (2015) VERSE: a novel approach to detect virus integration in host genomes through reference genome customization. The global health burden of infection‐associated cancers in the year 2002. Genome Med 7, 1–9. 25699093 10.1186/s13073-015-0126-6 PMC4333248

[46] Li H , Handsaker B , Wysoker A , Fennell T , Ruan J , Homer N , Marth G , Abecasis G , Durbin R , 1000 Genome Project Data Processing Subgroup (2009) The sequence alignment/Map format and SAMtools. Bioinformatics 25, 2078–2079. 19505943 10.1093/bioinformatics/btp352 PMC2723002

[47] Futreal PA , Coin L , Marshall M , Down T , Hubbard T , Wooster R , Rahman N & Stratton MR (2004) A census of human cancer genes. Nat Rev Cancer 4, 177–183. 14993899 10.1038/nrc1299 PMC2665285

[48] Kandoth C , McLellan MD , Vandin F , Ye K , Niu B , Lu C , Xie M , Zhang Q , McMichael JF , Wyczalkowski MA *et al*. (2013) Mutational landscape and significance across 12 major cancer types. Nature 502, 333–339. 24132290 10.1038/nature12634 PMC3927368

[49] Lawrence MS , Stojanov P , Mermel CH , Robinson JT , Garraway LA , Golub TR , Meyerson M , Gabriel SB , Lander ES & Getz G (2014) Discovery and saturation analysis of cancer genes across 21 tumour types. Nature 505, 495–501. 24390350 10.1038/nature12912 PMC4048962

[50] Smeets SJ , Braakhuis BJ , Abbas S , Snijders PJ , Ylstra B , van de Wiel MA , Meijer GA , Leemans CR & Brakenhoff RH (2006) Genome‐wide DNA copy number alterations in head and neck squamous cell carcinomas with or without oncogene‐expressing human papillomavirus. Oncogene 25, 2558–2564. 16314836 10.1038/sj.onc.1209275

[51] Ojesina AI , Lichtenstein L , Freeman SS , Pedamallu CS , Imaz‐Rosshandler I , Pugh TJ , Cherniack AD , Ambrogio L , Cibulskis K , Bertelsen B *et al*. (2014) Landscape of genomic alterations in cervical carcinomas. Nature 506, 371–375. 24390348 10.1038/nature12881 PMC4161954

[52] Peifer M , Fernandez‐Cuesta L , Sos ML , George J , Seidel D , Kasper LH , Plenker D , Leenders F , Sun R , Zander T *et al*. (2012) Integrative genome analyses identify key somatic driver mutations of small‐cell lung cancer. Nat Genet 44, 1104–1110. 22941188 10.1038/ng.2396 PMC4915822

[53] Stransky N , Egloff AM , Tward AD , Kostic AD , Cibulskis K , Sivachenko A , Kryukov GV , Lawrence MS , Sougnez C , McKenna A *et al*. (2011) The mutational landscape of head and neck squamous cell carcinoma. Science 333, 1157–1160. 21798893 10.1126/science.1208130 PMC3415217

[54] Alexandrov LB , Nik‐Zainal S , Wedge DC , Aparicio SA , Behjati S , Biankin AV , Bignell GR , Bolli N , Borg A , Borresen‐Dale AL *et al*. (2013) Signatures of mutational processes in human cancer. Nature 500, 415–421. 23945592 10.1038/nature12477 PMC3776390

[55] Chen S , Li X , Qin J , Chen Y , Liu L , Zhang D , Wang M , Wang M & Zhang D (2015) APOBEC3A possesses anticancer and antiviral effects by differential inhibition of HPV E6 and E7 expression on cervical cancer. Int J Clin Exp Med 8, 10548–10557. 26379844 PMC4565227

[56] Sanchez‐Vega F , Mina M , Armenia J , Chatila WK , Luna A , La KC , Dimitriadoy S , Liu DL , Kantheti HS , Saghafinia S *et al*. (2018) Oncogenic signaling pathways in the cancer genome atlas. Cell 173, 321–337.e310. 29625050 10.1016/j.cell.2018.03.035 PMC6070353

[57] Cancer Genome Atlas Network (2012) Comprehensive molecular portraits of human breast tumours. Nature 490, 61–70. 23000897 10.1038/nature11412 PMC3465532

[58] Cancer Genome Atlas Research Network , Kandoth C , Schultz N , Cherniack AD , Akbani R , Liu Y , Shen H , Robertson AG , Pashtan I , Shen R *et al*. (2013) Integrated genomic characterization of endometrial carcinoma. Nature 497, 67–73. 23636398 10.1038/nature12113 PMC3704730

[59] Wang NJ , Sanborn Z , Arnett KL , Bayston LJ , Liao W , Proby CM , Leigh IM , Collisson EA , Gordon PB , Jakkula L *et al*. (2011) Loss‐of‐function mutations in Notch receptors in cutaneous and lung squamous cell carcinoma. Proc Natl Acad Sci USA 108, 17761–17766. 22006338 10.1073/pnas.1114669108 PMC3203814

[60] Zagouras P , Stifani S , Blaumueller CM , Carcangiu ML & Artavanis‐Tsakonas S (1995) Alterations in Notch signaling in neoplastic lesions of the human cervix. Proc Natl Acad Sci USA 92, 6414–6418. 7604005 10.1073/pnas.92.14.6414 PMC41528

[61] Weng AP , Ferrando AA , Lee W , Morris JP 4th , Silverman LB , Sanchez‐Irizarry C , Blacklow SC , Look AT & Aster JC (2004) Activating mutations of NOTCH1 in human T cell acute lymphoblastic leukemia. Science 306, 269–271. 15472075 10.1126/science.1102160

[62] Yachida S , Vakiani E , White CM , Zhong Y , Saunders T , Morgan R , de Wilde RF , Maitra A , Hicks J , Demarzo AM *et al*. (2012) Small cell and large cell neuroendocrine carcinomas of the pancreas are genetically similar and distinct from well‐differentiated pancreatic neuroendocrine tumors. Am J Surg Pathol 36, 173–184. 22251937 10.1097/PAS.0b013e3182417d36 PMC3261427

[63] Zheng X , Liu D , Fallon JT & Zhong M (2015) Distinct genetic alterations in small cell carcinoma from different anatomic sites. Exp Hematol Oncol 4, 2. 25937998 10.1186/2162-3619-4-2 PMC4417281

[64] Travis WD (2012) Update on small cell carcinoma and its differentiation from squamous cell carcinoma and other non‐small cell carcinomas. Mod Pathol 25 (Suppl 1), S18–30. 22214967 10.1038/modpathol.2011.150

[65] Cho SY , Choi M , Ban HJ , Lee CH , Park S , Kim H , Kim YS , Lee YS & Lee JY (2017) Cervical small cell neuroendocrine tumor mutation profiles via whole exome sequencing. Oncotarget 8, 8095–8104. 28042953 10.18632/oncotarget.14098 PMC5352385

[66] Salvo G , Gonzalez Martin A , Gonzales NR & Frumovitz M (2019) Updates and management algorithm for neuroendocrine tumors of the uterine cervix. Int J Gynecol Cancer 29, 986–995. 31263021 10.1136/ijgc-2019-000504

[67] zur Hausen H (2002) Papillomaviruses and cancer: from basic studies to clinical application. Nat Rev Cancer 2, 342–350. 12044010 10.1038/nrc798

