# Supplementary material for: Genomic characterization of small cell carcinomas of the uterine cervix
Source: Mol Oncol. 2021 May 13;16(4):833–45. doi: 10.1002/1878-0261.12962 (PMC8847983; doi:10.1002/1878-0261.12962)
Supplement: Supplementary file 1 — Fig. S1. Comparison of whole‐exome and high‐depth targeted sequencing of uterine cervix small cell carcinomas SCC1 and SCC4. Fig. S2. Detection of HPV16 and HPV18 DNA in uterine cervix small cell carcinomas. Table S1. Antibody clones, dilutions, antigen retrievals and scoring used for the immunohistochemical analyses performed. Table S2. Primers for Sanger sequencing validation of mutations identified by whole‐exome sequencing. Table S3. Sequencing statistics, number of somatic mutations identified and validation rates. Table S4. Non‐synonymous somatic mutations identified in small cell carcinomas of the uterine cervix using whole‐exome and MSK‐IMPACT targeted sequencing. Table S5. Putative in‐frame fusion transcripts with driver probability > 0.1 identified in SCC1 and SCC2 using RNA‐sequencing. Table S6. Putative HPV integration sites based on RNA‐sequencing. [file MOL2-16-833-s001.pdf]

SUPPLEMENTARY MATERIALS

Genomic characterization of small cell carcinomas of the uterine cervix

Schultheis et al.

Supplementary Figures S1 and S2

Supplementary Tables S1 - S6

Supplementary Figure S1

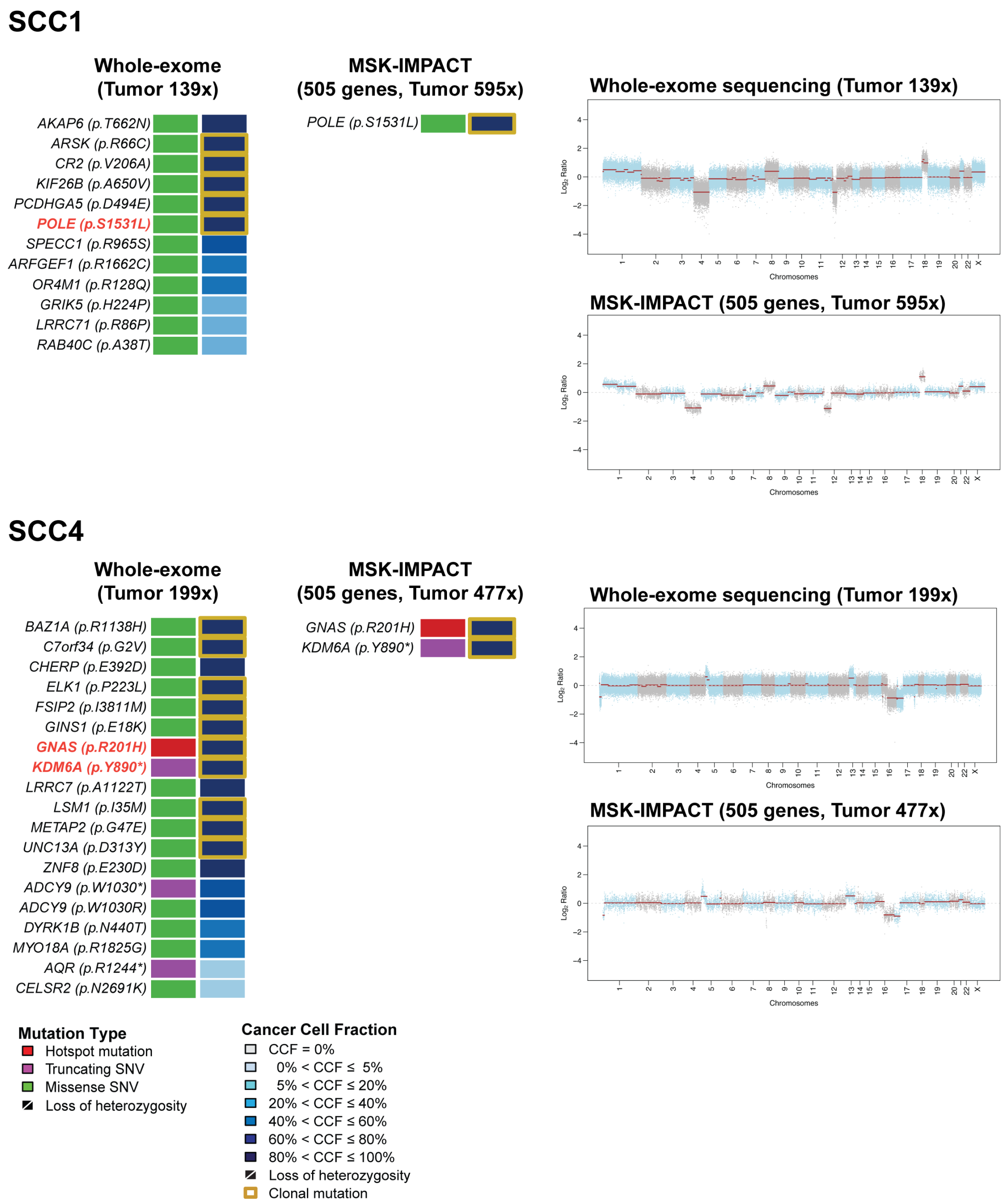

Supplementary Figure S1: Comparison of whole-exome and high-depth targeted sequencing of uterine cervix small cell carcinomas SCC1 and SCC4. The same tumor and normal derived DNA samples of SCC1 (top) and SCC4 (bottom) were subjected to whole-exome sequencing and MSK-IMPACT sequencing targeting 505 cancer-related genes. The mutations identified by whole-exome sequencing are shown on the left, and genes being part of the 505 MSK-IMPACT panel are labeled in an orange font. All mutations identified by whole-exome sequencing affecting genes that were part of the 505 targeted panel were identified by MSK-IMPACT sequencing (middle), and no additional mutations were found. The copy number profiles of SCC1 and SCC4 based on whole-exome sequencing and MSK-IMPACT sequencing are also comparable.

Supplementary Figure S2

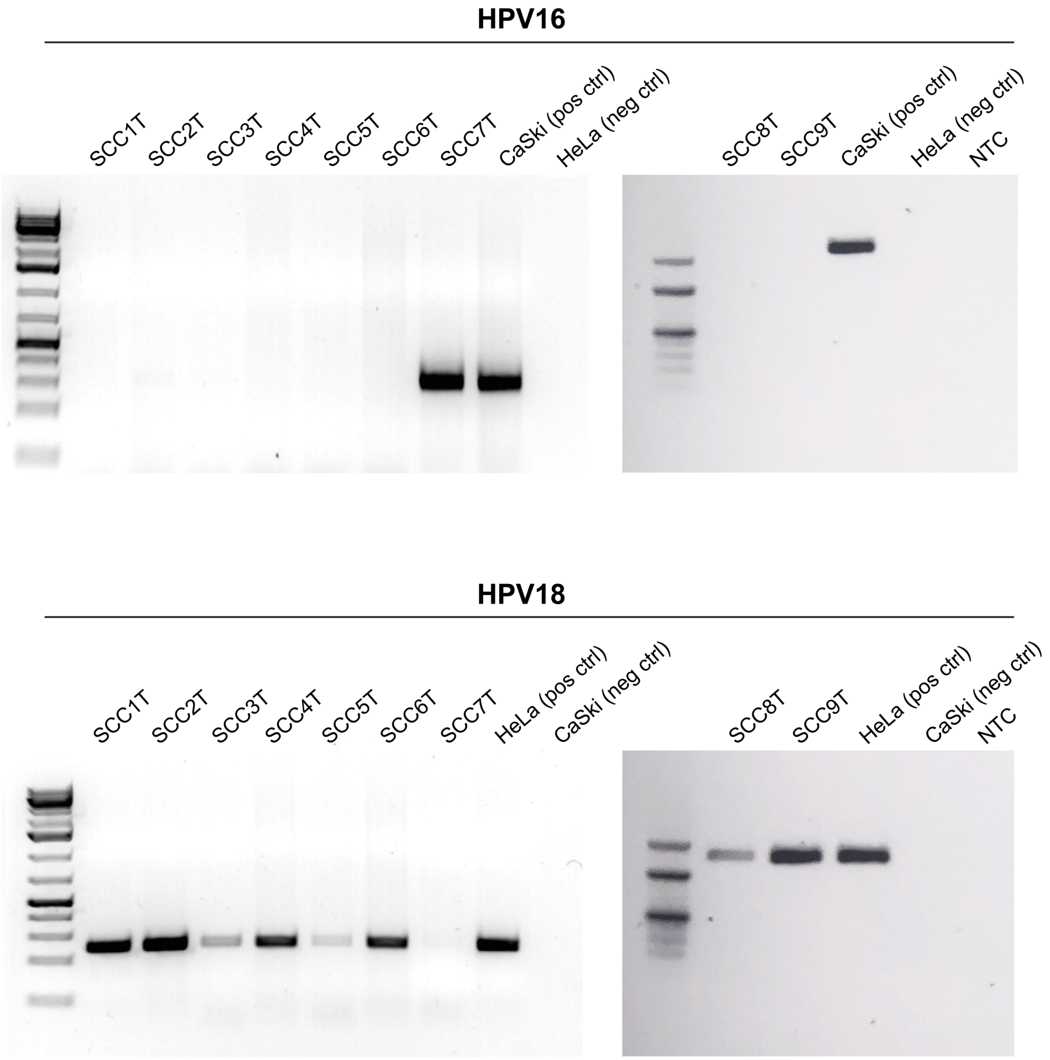

Supplementary Figure S2. Detection of HPV16 and HPV18 DNA in uterine cervix small cell carcinomas. DNA from uterine cervix small cell carcinomas was amplified with HPV16-specific primers (top) or with HPV18-specific primers (bottom). Agarose gel electrophoresis is shown. CaSki- and HeLa-derived cell line DNA, which are known to harbor HPV16 and HPV18, respectively, were included as positive controls. Ctrl, control. NTC, no template control.

Supplementary Table S1. Antibody clones, dilutions, antigen retrievals and scoring used for the immunohistochemical analyses performed.

Antibody	Clone	Dilution	Antigen retrieval	Detection	Company	Scoring
Ki67	MIB1	1:400	ER2, 30’	Leica Bond-RX-BOND Polymer Refine Detection	DAKO	Nuclear positivity in tumor cells; percentage counting 500 tumor cell nuclei.
p63	4A4	RTU	CC1, 24’	Ventana - OptiView	Ventana	No nuclear positivity allowed in the tumor cells.
Chromo-granin A	LK2H10	RTU	CC1, 8'	Ventana - Optiview	Ventana	Positive cytoplasmic staining in tumor cells at any percentage.
Synapto-physin	Snp88	1:2000	CC1, 32'	Ventana - Optiview	Biogenex	Positive cytoplasmic staining in tumor cells at any percentage.
p16	E6H4	RTU	CC1, 32'	Ventana - Optiview	Ventana	Nuclear positivity and cytoplasmic positivity in tumor cells.
Pan-cytokeratin	Clone AE1/AE3	1:600	ER2, 30’	Ventana - Optiview	DAKO	Cytoplasmic positivity in tumor cells at any percentage considered as positive

RTU, ready to use.

Supplementary Table S2. Primers for Sanger sequencing validation of mutations identified by whole-exome sequencing.

Sample	Gene	AA change	Chromosome	Position	Forward	Reverse
SCC1T	AKAP6	T662N	14	3208695	GAATACCTAGCACTGCCCTCTC	CCTGCCTAGCCTTGTATGCT
SCC1T	ARSK	R66C	5	94901771	ATCAATGCTAATTAAATTTCTTTATTTCC	TGTAGCCACTTTCTCCCATTC
SCC1T	KIF26B	A650V	1	245775129	CCCAGGCATGAGTGACAG	GAAGAACACGTGTGAGTTGCG
SCC1T	POLE	S1531L	12	133219542	AATCGCTGGATGGCTCTG	CAGTGGTGGAGTCTGCCC
SCC1T	SPECC1	R965S	17	20163560	AGTATTAGCATCTTCTCCTTACCATGT	CAGGACTACTACTGCCCAGGG
SCC2T	AGA	E130Q	4	178360736	CTTAGATCTGGTTTTATCCATGATTTT	TATGGATGTAGGAGCAGTAGGAGA
SCC2T	ARSF	C151*	X	3002330	TCACATGGTTTTTGCAATGTG	GAGCCAGAGCTGACTCTCAAA
SCC2T	BDP1	Q1353*	5	70806976	GGAAAACGAGCTAGAGGAGACC	TAAGTGTTGTTGGACTTAAGGACAAA
SCC2T	COL5A1	R498S	9	137630654	TTGGACCTTGCCCTGC	TGAAGACACAAGCCTCTGGG
SCC2T	GLI3	R905H	7	42005957	GTCTTCAGGCTCATCCTCTCC	GCTCGGCCTACCTGAGCA
SCC2T	OR2D3	R138W	11	6942644	GAGGAAAACCATTTCTTTTTATGG	ACTAGTGCCCCACTGGCC
SCC2T	PIK3CA	E545K	3	178936091	AGGGGAAAAATATGACAAAGAAAG	CTGCTTTATTTATTCCAATAGGTATGG
SCC2T	SNRNP200	R1090W	2	96953698	TACATGCGTTTGTCGATCATC	GAGCTGGAGGTAATGAAACCG
SCC2T	TP53	R175H	17	7578406	AGACCTAAGAGCAATCAGTGAGGA	GCCAACTGGCCAAGACCT
SCC2T	UTP20	L583S	12	101696298	GTGGTTTCTCCTGTTTTTCTGC	GATTTCACAACAATAGAGGCTTTT
SCC2T	ZNF584	E378K	19	58929017	CTGGAAAGTCCACACTGGG	TCCTGACGCCTTTCTGGA
SCC4T	CHERP	E392D	19	16639020	GTGGGATCTGGTCGTGTG	TAACTGCCTGTCACTTGGATTTAG
SCC4T	MYO18A	R1825G	15	72208836	GACTTGGTAATGCGATCTTGTAGT	GAAAACTGGCTGAGAATTTTGG
SCC4T	LRRC7	A1122T	1	70504985	TTCTCAGCCATCTGTGAATGAG	CGATCCAGCCCTCCCCTATA
SCC4T	GINS1	E18K	20	25388508	CGCCATGTTCTGCGAAA	TGCCCCGTCCAAGTCTAC
SCC4T	BAZ1A	R1138H	14	35234363	GACATATCTTGCAACGCGC	TTCTTCTGCTAGTCTATCCCAAGTTT
SCC4T	METAP2	G47E	12	95868095	GAAGAAGGAGCTGCCTCTACG	TGAGCCAAACAGTTCAGCC
SCC4T	KDM6A	Y890*	X	44929570	AAACAGATCTGCTTCTGGTTAACC	TCAAACATTTTCACTTTGATGAACTT
SCC4T	ELK1	P223L	X	47497568	CTCTTCCTTGGGCCCTTCTA	CCCGGGGAAATCACTG
SCC4T	ADCY9	W1030*	16	4016748	GGTTCTTGGAGTAGGTCTGGG	ACTACCACGGAGACGTGGAA
SCC4T	ADCY9	W1030R	16	4016748	GGTTCTTGGAGTAGGTCTGGG	ACTACCACGGAGACGTGGAA
SCC4T	GNAS	R201H	20	57484421	TATTACTGTTTCGGTTGGCTTTG	ACAGTTGGCTTACTGGAAGTTGA
SCC4T	UNC13A	D313Y	19	17767038	TCCTCCTCCTCCAGGAAGTC	CTCCGACATGGAGGATGAG
SCC5T	ANKRD44	P945S	2	197858397	CTGCAGTCGCTGTTGACTTT	ATCCATGTACTATTTGTAGAGTCTAAGTAC
SCC5T	ROBO2	W451C	3	77607216	TTTGACAGATAGACCTCCACCTATAA	AGATGATAATTCATGTACAAGTCCAAT
SCC5T	RQCD1	R259H	2	219457379	AGAATTTGTTTGTGTTTTGTGTGTTT	GTTCAGCAGAGTATTTCCCTAAAGTC
SCC5T	FAM120A	T996S	9	96324527	TTCATTCATTTAGGCGTCCAA	TGCCTGGTTTTTGTAACCTCT
SCC5T	FLNC	R1008H	7	128484151	GTGAACACACGAGGGGCT	TAGCGCACAGCCTGGG
SCC5T	PCDH17	L48F	13	58206822	TGCCCTCACTCTCAAGAACC	CTGTCTGCGTCCACGTCC
SCC5T	ONECUT1	P249L	15	53081336	ATTGCTGACCTGCGCG	AACGGCTTCGAAGCCC
SCC5T	CHAT	Q311P	10	50833698	GACACGCTGGTGGCTCAG	GTAATGGACGTGAACGCATG
SCC5T	FZR1	M27V	19	3525875	CTGGGGGCACTCTCGG	GGGATGAAGCGGTCTCC
SCC5T	SCIN	V122M	7	12620694	GGCAGACACCTCATCAGTTTT	CGACCCTTCACATGTAGGAG
SCC5T	ITSN1	P113S	21	35107500	CATAGCTATGAAACTTATCAAACTGAA	ATAATGTTATAAACCGAGAGGGAAAA
SCC5T	PLA2G4F	F318I	15	42442018	TGCTGCAGGGCCTTG	CACCCAGAACCCCCCA
SCC5T	CACNA1F	G985E	X	49074921	TGATCCTGGCCCCAGG	ATCTCGGTGGTGAAGATTCTG
SCC6T	IDH1	H309Y	2	209104653	GGGCTGCTTTGGAGAGCA	CTCTTCATGCAGTTGGACCC
SCC6T	LAMB4	R1252T	7	107696077	ATCAGTCCCCTAAAGTCAACATAGG	TCTGTGAGGCAGACTTCAAAGA
SCC6T	METTL18	M1I	1	169762834	AGAACATTTTCTGTCCCTCTCTTCT	CCATGTATAAGCTGCTAAAGAGAAATT
SCC6T	NIPBL	S2013I	5	37038770	GTTTCCACTACCTTGTCATATTTTAGTG	CTAAATTTAAAGCACTTAATGTTTTATGT
SCC6T	NOTCH2	E1025K	1	120483288	AGTCAATTACCTGACAGTTTTTCCC	TAGCTGATGTTGCTCTGTTTGC
SCC6T	NPY5R	F78L	4	164271657	GATGACTTACAGTATTTTCTGATTGGG	CATAATATGGCACATGACTTTGC
SCC6T	ZCCHC11	D237N	1	52991244	GTAATTAAAATGGTTAAACCGGGG	TGCAAAACTCACCACGATCT
SCC6T	ZCCHC8	Q205*	12	122967884	AAGCCAAAAGTTTATGATTTTGGT	CTTTCCGAAGGATGGGAAA
SCC6T	RP11-1220K2.2	R356C	7	141838447	TCTTGGGTTCCAGCTTAGTCG	TAAAAATGGGAATTGGTGTCAAC
SCC7T	C19orf57	E245*	19	14000936	CAGGGGACACCGTCTCC	CCTGTCAGAACAAGGGGC
SCC7T	CEP97	Q566E	3	101477146	AAAGAAACCATATCTCAAGCAACTTC	TCCTTATTTCATCAGTTAAGCAGACA
SCC7T	CEP97	S330*	3	101476002	TAGACCAGGTATTATTGATTTTTAGGT	ATATGCACCTTTTTCTGCTTTATGA
SCC7T	CEP97	Q580*	3	101477188	ATTTTAACCCAGAGATCTGTTGCT	CAAACCAACAAAACAGTGCATC
SCC7T	FGFR3	E140K	4	1801512	GGACCCTGCCCCATCT	CCTTAGTCCCTCAGCTGCC
SCC7T	GBE1	H291Q	3	81692051	TGCTATCCCAAAGATCATGAGTC	TCATTGTTAGCCGTTATGGAAC
SCC7T	IL24	E105K	1	207074845	GAAGATCCCTATCTCTGCTGTG	CCAGAGTAGAGAATGACTTCAGAGTC
SCC7T	KANK4	P795T	1	62728920	AGTGGGACACGCTGTAGTGA	GGAGGAACTCTTGCTGGC
SCC7T	NLRP14	C505*	11	7064772	ATGAAAACTGCTATGTGTTCACCC	CTTCATTCAAAAGGCCAAACA
SCC7T	P2RX7	E175Q	12	121600313	TTTGACCCCTATAGGAATTCAGAC	CTGAGAGGGGAAGGCCC
SCC7T	RNF168	Q105*	3	196215543	ACTAAATTAAAATCTGAAACTAAAAACACA	CAAAAGGAGATATTGCTGCAGTT
SCC7T	SLC12A7	E989Q	5	1057647	AGGATCTGTTCCCCCCAG	CAGGAACACCGCGTCC
SCC7T	TMEM51	L213F	1	15546114	TCACTGACGGGGCTCG	CGTGGCTCAAGTGGGG
SCC7T	INPP5D	L46Q	2	234104059	CTGCAAATGGAAACCCCTT	ACCCCACGCCCACTTTAC
SCC7T	CACNA1A	R1977H	19	13325057	GTGGTGACATGCAAGCCA	AAGATCTACGCAGCCATGATG

Supplementary Table S3. Sequencing statistics, number of somatic mutations and validation rates.

Case	Tissue Type	Sequencing Type	Mean Target Coverage (X)	Target Bases 2X	Target Bases 50X	Target Bases 100X	Total # Somatic Mutations	# Somatic Non-synonymous Mutations	Validation Rate Sanger Sequencing	Validation Rate MSK-IMPACT
**SCC1**	Normal	Whole-exome	88	100%	77%	34%	21	12	100% (5/5)	100% (1/1)
	Tumor		139	100%	92%	68%				
**SCC2**	Normal	Whole-exome	121	100%	98%	93%	34	24	100% (11/11)	Not performed
	Tumor		336	100%	90%	59%				
**SCC3**	Normal	MSK-IMPACT 505	122	95%	78%	56%	10	6	Not performed	Not performed
	Tumor		408	100%	99%	99%				
**SCC4**	Normal	Whole-exome	152	100%	96%	84%	32	19 (18 validated)	92% (11/12)	100% (2/2)
	Tumor		199	100%	95%	75%				
**SCC5**	Normal	Whole-exome	140	100%	97%	88%	41	26	100% (13/13)	Not performed
	Tumor		221	100%	93%	69%				
**SCC6**	Normal	Whole-exome	181	100%	93%	73%	42	26	100% (9/9)	Not performed
	Tumor		182	100%	96%	80%				
**SCC7**	Normal	Whole-exome	175	100%	91%	70%	84	59 (57 validated)	87% (13/15)	Not performed
	Tumor		183	100%	94%	75%				
**SCC8**	Normal Pool	MSK-IMPACT 505	115	100%	79%	55%	0	0	Not applicable	Not performed
	Tumor		278	99%	99%	97%				
**SCC9**	Normal	MSK-IMPACT 505	202	100%	99%	93%	10	8	Not performed	Not performed
	Tumor		405	100%	99%	99%				

Supplementary Table S4. Non-synonymous somatic mutations identified in small cell carcinomas of the cervix using whole-exome or MSK-IMPACT targeted sequencing.

Sample ID	Sequencing type	Chromosome	Position	Reference	Alternate	Gene symbol	AA change	Mutation type	Tumor MAF	Normal MAF	LOH	Pathogenicity	Hotspot mutation	Cancer cell fraction	Clonal status	RNASeq Validation	Sanger validation
SCC1T	WES	14	33015844	C	A	AKAP6	p.T662N	Missense_Mutation	0.4137931	0.004	No	passenger	No	0.83	Subclonal	Mutation Expressed	Validated
SCC1T	WES	8	68115462	G	A	ARFGEF1	p.R1662C	Missense_Mutation	0.14545455	0	No	passenger	No	0.44	Subclonal	Not Expressed	Not Tested
SCC1T	WES	5	94901771	C	T	ARSK	p.R66C	Missense_Mutation	0.515625	0.01290323	No	likely_pathogenic	No	1	Clonal	Not Expressed	Validated
SCC1T	WES	1	207642043	T	C	CR2	p.V206A	Missense_Mutation	0.288	0.00843882	No	passenger	No	0.86	Clonal	Not Expressed	Not Tested
SCC1T	WES	19	42563517	T	G	GRIK5	p.H224P	Missense_Mutation	0.13157895	0	No	likely_pathogenic	No	0.26	Subclonal	NA	Not Tested
SCC1T	WES	1	245775129	C	T	KIF26B	p.A650V	Missense_Mutation	0.5	0	No	passenger	No	1	Clonal	Mutation Expressed	Validated
SCC1T	WES	1	156893837	G	C	LRRC71	p.R86P	Missense_Mutation	0.125	0	No	passenger	No	0.38	Subclonal	NA	Not Tested
SCC1T	WES	14	20248864	G	A	OR4M1	p.R128Q	Missense_Mutation	0.27272727	0.00452489	No	passenger	No	0.55	Subclonal	Not Expressed	Not Tested
SCC1T	WES	5	140745379	C	A	PCDHGA5	p.D494E	Missense_Mutation	0.51449275	0	No	passenger	No	1	Clonal	Not Expressed	Not Tested
SCC1T	WES	12	133219542	G	A	POLE	p.S1531L	Missense_Mutation	0.5	0.0483871	No	passenger	No	1	Clonal	Mutation Expressed	Validated
SCC1T	WES	16	640403	G	A	RAB40C	p.A38T	Missense_Mutation	0.14893617	0.02702703	No	passenger	No	0.3	Subclonal	NA	Not Tested
SCC1T	WES	17	20163560	C	A	SPECC1	p.R965S	Missense_Mutation	0.34615385	0	No	passenger	No	0.69	Subclonal	Mutation Expressed	Validated
SCC2T	WES	11	76572040	G	A	ACER3	p.R7Q	Missense_Mutation	0.51724138	0	No	passenger	No	1	Clonal	Not Expressed	Not Tested
SCC2T	WES	4	178360736	C	G	AGA	p.E130Q	Missense_Mutation	0.43939394	0	No	likely_pathogenic	No	0.97	Clonal	Mutation Expressed	Validated
SCC2T	WES	23	3002330	C	A	ARSF	p.C151*	Nonsense_Mutation	0.46082949	0	No	passenger	No	1	Clonal	Not Expressed	Validated
SCC2T	WES	16	71885349	A	G	ATXN1L	p.H569R	Missense_Mutation	0.45016077	0	No	passenger	No	0.99	Clonal	NA	Not Tested
SCC2T	WES	5	70806976	C	T	BDP1	p.Q1353*	Nonsense_Mutation	0.44162437	0	No	passenger	No	0.98	Clonal	Mutation Expressed	Validated
SCC2T	WES	6	36169471	A	C	BRPF3	p.K458Q	Missense_Mutation	0.09473684	0	No	passenger	No	0.3	Subclonal	Mutation Expressed	Not Tested
SCC2T	WES	19	41255672	T	G	C19orf54	p.I13L	Missense_Mutation	0.4611399	0.01538462	No	passenger	No	1	Clonal	Mutation Expressed	Not Tested
SCC2T	WES	14	105350729	T	TG	CEP170B	p.T542Dfs*25	Frame_Shift_Ins	0.21167883	0	No	passenger	No	0.47	Subclonal	NA	Not Tested
SCC2T	WES	9	137630654	G	T	COL5A1	p.R498S	Missense_Mutation	0.41296928	0	No	likely_pathogenic	No	0.91	Clonal	Not Expressed	Validated
SCC2T	WES	11	66335541	A	C	CTSF	p.S76A	Missense_Mutation	0.19642857	0	No	passenger	No	0.43	Subclonal	Not Expressed	Not Tested
SCC2T	WES	10	96612523	G	A	CYP2C19	p.R442H	Missense_Mutation	0.08247423	0	No	passenger	No	0.18	Subclonal	Not Expressed	Not Tested
SCC2T	WES	7	42005957	C	T	GLI3	p.R905H	Missense_Mutation	0.4	0	No	likely_pathogenic	No	0.88	Clonal	Not Expressed	Validated
SCC2T	WES	2	97274642	A	G	KANSL3	p.S515P	Missense_Mutation	0.1	0.01666667	No	passenger	No	0.22	Subclonal	NA	Not Tested
SCC2T	WES	11	6942644	C	T	OR2D3	p.R138W	Missense_Mutation	0.45054945	0	No	passenger	No	1	Clonal	Not Expressed	Validated
SCC2T	WES	3	178936091	G	A	PIK3CA	p.E545K	Missense_Mutation	0.45631068	0.00735294	No	likely_pathogenic	Yes	1	Clonal	Mutation Expressed	Validated
SCC2T	WES	1	109964531	G	A	PSMA5	p.S16F	Missense_Mutation	0.13636364	0.00980392	No	passenger	No	0.3	Subclonal	NA	Not Tested
SCC2T	WES	8	52387648	T	C	PXDNL	p.E193G	Missense_Mutation	0.15625	0	No	passenger	No	0.5	Subclonal	Not Expressed	Not Tested
SCC2T	WES	16	67995498	T	G	SLC12A4	p.T110P	Missense_Mutation	0.16504854	0.02083333	No	passenger	No	0.36	Subclonal	NA	Not Tested
SCC2T	WES	2	96953698	G	A	SNRNP200	p.R1090W	Missense_Mutation	0.43176734	0	No	passenger	No	0.95	Clonal	Mutation Expressed	Validated
SCC2T	WES	16	30748409	AAGG	A	SRCAP	p.E2352del	In_Frame_Del	0.55555556	0	No	passenger	No	1	Clonal	Mutation Expressed	Not Tested
SCC2T	WES	17	7578406	C	T	TP53	p.R175H	Missense_Mutation	0.51117318	0	No	likely_pathogenic	Yes	1	Clonal	Mutation Expressed	Validated
SCC2T	WES	2	128945090	A	G	UGGT1	p.D1515G	Missense_Mutation	0.17037037	0.01428571	No	passenger	No	0.38	Subclonal	Mutation Expressed	Not Tested
SCC2T	WES	12	101696298	T	C	UTP20	p.L583S	Missense_Mutation	0.45327103	0	No	passenger	No	1	Clonal	Mutation Expressed	Validated
SCC2T	WES	19	58929017	G	A	ZNF584	p.E378K	Missense_Mutation	0.44369369	0	No	passenger	No	0.98	Clonal	Mutation Expressed	Validated
SCC4T	WES	16	4016750	A	T	ADCY9	p.W1030R	Missense_Mutation	0.36526946	0	No	likely_pathogenic	No	0.77	Subclonal	RNASeq not available	Validated
SCC4T	WES	16	4016748	C	T	ADCY9	p.W1030*	Nonsense_Mutation	0.36746988	0	No	passenger	No	0.77	Subclonal	RNASeq not available	Validated
SCC4T	WES	15	35162991	G	A	AQR	p.R1244*	Nonsense_Mutation	0.07407407	0.00840336	No	passenger	No	0.16	Subclonal	RNASeq not available	Not Tested
SCC4T	WES	14	35234363	C	T	BAZ1A	p.R1138H	Missense_Mutation	0.55102041	0	No	likely_pathogenic	No	1	Clonal	RNASeq not available	Validated
SCC4T	WES	7	142636648	G	T	C7orf34	p.G2V	Missense_Mutation	0.55172414	0	No	passenger	No	1	Clonal	RNASeq not available	Not Tested
SCC4T	WES	1	109815280	C	A	CELSR2	p.N2691K	Missense_Mutation	0.06521739	0	No	passenger	No	0.14	Subclonal	RNASeq not available	Not Tested
SCC4T	WES	19	16639020	T	G	CHERP	p.E392D	Missense_Mutation	0.39090909	0	No	passenger	No	0.82	Subclonal	RNASeq not available	Validated
SCC4T	WES	19	40317404	T	G	DYRK1B	p.N440T	Missense_Mutation	0.2247191	0	No	passenger	No	0.47	Subclonal	RNASeq not available	Not Tested
SCC4T	WES	23	47497568	G	A	ELK1	p.P223L	Missense_Mutation	0.43076923	0	No	passenger	No	0.91	Clonal	RNASeq not available	Validated
SCC4T	WES	2	186665199	A	G	FSIP2	p.I3811M	Missense_Mutation	0.51086957	0	No	passenger	No	1	Clonal	RNASeq not available	Not Tested
SCC4T	WES	20	25388508	G	A	GINS1	p.E18K	Missense_Mutation	0.58974359	0	No	passenger	No	1	Clonal	RNASeq not available	Validated
SCC4T	WES	20	57484421	G	A	GNAS	p.R201H	Missense_Mutation	0.44117647	0	No	likely_pathogenic	Yes	0.93	Clonal	RNASeq not available	Validated
SCC4T	WES	23	44929570	C	A	KDM6A	p.Y890*	Nonsense_Mutation	0.49673203	0	No	likely_pathogenic	No	1	Clonal	RNASeq not available	Validated
SCC4T	WES	1	70504985	G	A	LRRC7	p.A1122T	Missense_Mutation	0.40993789	0.00704225	No	passenger	No	0.86	Subclonal	RNASeq not available	Validated
SCC4T	WES	8	38029493	A	C	LSM1	p.I35M	Missense_Mutation	0.45794393	0	No	passenger	No	0.96	Clonal	RNASeq not available	Not Tested
SCC4T	WES	12	95868095	G	A	METAP2	p.G47E	Missense_Mutation	0.47507331	0	No	passenger	No	1	Clonal	RNASeq not available	Validated
SCC4T	WES	17	27417032	G	C	MYO18A	p.R1825G	Missense_Mutation	0.19310345	0	No	passenger	No	0.41	Subclonal	RNASeq not available	Not Validated
SCC4T	WES	19	17767038	C	A	UNC13A	p.D313Y	Missense_Mutation	0.43703704	0	No	likely_pathogenic	No	0.92	Clonal	RNASeq not available	Validated
SCC4T	WES	19	58805864	A	T	ZNF8	p.E230D	Missense_Mutation	0.39041096	0	No	passenger	No	0.82	Subclonal	RNASeq not available	Not Tested
SCC5T	WES	2	197858397	G	A	ANKRD44	p.P937S	Missense_Mutation	0.44	0	No	passenger	No	0.97	Clonal	RNASeq not available	Validated
SCC5T	WES	10	33137556	G	A	C10ORF68	p.V512I	Missense_Mutation	0.48837209	0	No	passenger	No	1	Clonal	RNASeq not available	Not Tested
SCC5T	WES	8	140944767	A	G	C8orf17	p.K80E	Missense_Mutation	0.08064516	0	No	passenger	No	0.18	Subclonal	RNASeq not available	Not Tested
SCC5T	WES	23	49074921	C	T	CACNA1F	p.G985E	Missense_Mutation	0.29166667	0	No	likely_pathogenic	No	0.93	Clonal	RNASeq not available	Validated
SCC5T	WES	10	50833698	A	C	CHAT	p.Q311P	Missense_Mutation	0.48148148	0	No	likely_pathogenic	No	1	Clonal	RNASeq not available	Validated
SCC5T	WES	6	38893886	G	A	DNAH8	p.G3456D	Missense_Mutation	0.35238095	0	No	passenger	No	1	Clonal	RNASeq not available	Not Tested
SCC5T	WES	9	96324527	A	T	FAM120A	p.T996S	Missense_Mutation	0.50344828	0	No	passenger	No	1	Clonal	RNASeq not available	Validated
SCC5T	WES	7	128484151	G	A	FLNC	p.R1008H	Missense_Mutation	0.50862069	0	No	likely_pathogenic	No	1	Clonal	RNASeq not available	Validated
SCC5T	WES	19	3525875	A	G	FZR1	p.M27V	Missense_Mutation	0.41216216	0	No	passenger	No	1	Clonal	RNASeq not available	Validated
SCC5T	WES	10	118434512	T	C	HSPA12A	p.Y603C	Missense_Mutation	0.41276596	0.0030303	No	passenger	No	0.91	Clonal	RNASeq not available	Not Tested
SCC5T	WES	21	35107500	C	T	ITSN1	p.P113S	Missense_Mutation	0.40594059	0.00546448	No	passenger	No	0.89	Clonal	RNASeq not available	Validated
SCC5T	WES	1	204939764	G	A	NFASC	p.E342K	Missense_Mutation	0.3902439	0	No	passenger	No	0.86	Clonal	RNASeq not available	Not Tested
SCC5T	WES	15	53081336	G	A	ONECUT1	p.P249L	Missense_Mutation	0.42758621	0	No	passenger	No	0.94	Clonal	RNASeq not available	Validated
SCC5T	WES	13	58206822	C	T	PCDH17	p.L48F	Missense_Mutation	0.48251748	0	No	passenger	No	1	Clonal	RNASeq not available	Validated
SCC5T	WES	18	10671571	G	A	PIEZO2	p.R2738C	Missense_Mutation	0.47794118	0	No	likely_pathogenic	No	1	Clonal	RNASeq not available	Not Tested
SCC5T	WES	15	42442018	A	T	PLA2G4F	p.F318I	Missense_Mutation	0.4	0	No	passenger	No	0.88	Clonal	RNASeq not available	Validated
SCC5T	WES	3	38050807	G	A	PLCD1	p.A542V	Missense_Mutation	0.504	0	No	passenger	No	1	Clonal	RNASeq not available	Not Tested
SCC5T	WES	8	27312109	C	T	PTK2B	p.P932L	Missense_Mutation	0.08571429	0	No	likely_pathogenic	No	0.19	Subclonal	RNASeq not available	Not Tested
SCC5T	WES	23	135957528	T	A	RBMX	p.D224V	Missense_Mutation	0.41269841	0	No	passenger	No	1	Clonal	RNASeq not available	Not Tested
SCC5T	WES	3	77607216	G	C	ROBO2	p.W467C	Missense_Mutation	0.43529412	0	No	likely_pathogenic	No	0.96	Clonal	RNASeq not available	Validated
SCC5T	WES	2	219457379	G	A	RQCD1	p.R227H	Missense_Mutation	0.42372881	0	No	passenger	No	0.93	Clonal	RNASeq not available	Validated
SCC5T	WES	7	12620694	G	A	SCIN	p.V122M	Missense_Mutation	0.40625	0.02631579	No	passenger	No	0.89	Clonal	RNASeq not available	Validated
SCC5T	WES	6	152737801	A	T	SYNE1	p.V1924D	Missense_Mutation	0.64262295	0	No	passenger	No	1	Clonal	RNASeq not available	Not Tested
SCC5T	WES	23	67718915	G	C	YIPF6	p.E3Q	Missense_Mutation	0.31818182	0	No	passenger	No	1	Clonal	RNASeq not available	Not Tested
SCC5T	WES	19	19825209	C	G	ZNF14	p.D31H	Missense_Mutation	0.31770833	0	No	passenger	No	1	Clonal	RNASeq not available	Not Tested
SCC5T	WES	19	58187881	C	A	ZSCAN4	p.T123N	Missense_Mutation	0.26600985	0	No	passenger	No	0.85	Clonal	RNASeq not available	Not Tested
SCC6T	WES	3	49694742	G	A	BSN	p.V2585M	Missense_Mutation	0.23684211	0.0077821	No	passenger	No	0.85	Clonal	RNASeq not available	Not Tested
SCC6T	WES	5	41149571	C	A	C6	p.D799Y	Missense_Mutation	0.27272727	0	No	passenger	No	0.98	Clonal	RNASeq not available	Not Tested
SCC6T	WES	3	48677960	C	T	CELSR3	p.V3020M	Missense_Mutation	0.1958042	0	No	passenger	No	0.7	Subclonal	RNASeq not available	Not Tested
SCC6T	WES	1	222717222	C	T	HHIPL2	p.E211K	Missense_Mutation	0.24497992	0	No	passenger	No	1	Clonal	RNASeq not available	Not Tested
SCC6T	WES	6	26216735	A	T	HIST1H2BG	p.L46Q	Missense_Mutation	0.29032258	0	No	passenger	No	1	Clonal	RNASeq not available	Not Tested
SCC6T	WES	2	209104653	G	A	IDH1	p.H309Y	Missense_Mutation	0.38888889	0	No	likely_pathogenic	No	1	Clonal	RNASeq not available	Validated
SCC6T	WES	5	52218713	A	C	ITGA1	p.E800A	Missense_Mutation	0.1010101	0	No	passenger	No	0.36	Subclonal	RNASeq not available	Not Tested
SCC6T	WES	17	39135243	C	T	KRT40	p.E337K	Missense_Mutation	0.24489796	0	No	likely_pathogenic	No	1	Clonal	RNASeq not available	Not Tested
SCC6T	WES	7	107696077	C	G	LAMB4	p.R1252T	Missense_Mutation	0.49180328	0	No	passenger	No	1	Clonal	RNASeq not available	Validated
SCC6T	WES	8	86042196	G	A	LRRCC1	p.D557N	Missense_Mutation	0.2195122	0	No	passenger	No	1	Clonal	RNASeq not available	Not Tested
SCC6T	WES	1	169762834	C	T	METTL18	p.M1?	Translation_Start_Site	0.42857143	0	No	passenger	No	1	Clonal	RNASeq not available	Validated
SCC6T	WES	18	47479662	C	T	MYO5B	p.E574K	Missense_Mutation	0.2611465	0.00746269	No	likely_pathogenic	No	1	Clonal	RNASeq not available	Not Tested
SCC6T	WES	5	37038770	G	T	NIPBL	p.S2013I	Missense_Mutation	0.75159236	0	No	likely_pathogenic	No	1	Clonal	RNASeq not available	Validated
SCC6T	WES	1	120483288	C	T	NOTCH2	p.E1025K	Missense_Mutation	0.5	0	No	likely_pathogenic	No	1	Clonal	RNASeq not available	Validated
SCC6T	WES	4	164271657	T	C	NPY5R	p.F78L	Missense_Mutation	0.41428571	0	Yes	passenger	No	1	Clonal	RNASeq not available	Validated
SCC6T	WES	4	48852087	C	G	OCIAD1	p.S122C	Missense_Mutation	0.41111111	0	Yes	passenger	No	1	Clonal	RNASeq not available	Not Tested
SCC6T	WES	11	4703506	C	T	OR51E2	p.V146M	Missense_Mutation	0.04639175	0.00456621	No	passenger	No	0.12	Subclonal	RNASeq not available	Not Tested
SCC6T	WES	11	74053616	G	A	PGM2L1	p.R508C	Missense_Mutation	0.075	0	No	passenger	No	0.19	Subclonal	RNASeq not available	Not Tested
SCC6T	WES	7	141838447	C	T	RP11-1220K2.2	p.R356C	Missense_Mutation	0.21782178	0	No	passenger	No	0.78	Clonal	RNASeq not available	Validated
SCC6T	WES	9	19550147	G	C	SLC24A2	p.D489E	Missense_Mutation	0.19607843	0	No	passenger	No	0.7	Subclonal	RNASeq not available	Not Tested
SCC6T	WES	10	108371694	C	T	SORCS1	p.R1003Q	Missense_Mutation	0.25	0	No	passenger	No	0.9	Clonal	RNASeq not available	Not Tested
SCC6T	WES	5	14330980	C	G	TRIO	p.R609G	Missense_Mutation	0.08307692	0	No	passenger	No	0.71	Subclonal	RNASeq not available	Not Tested
SCC6T	WES	1	52991244	C	T	ZCCHC11	p.D237N	Missense_Mutation	0.32142857	0	No	likely_pathogenic	No	1	Clonal	RNASeq not available	Validated
SCC6T	WES	12	122967884	G	A	ZCCHC8	p.Q205*	Nonsense_Mutation	0.41981132	0.00471698	No	passenger	No	1	Clonal	RNASeq not available	Validated
SCC6T	WES	16	88500090	C	T	ZNF469	p.T2043I	Missense_Mutation	0.85333333	0	Yes	passenger	No	1	Clonal	RNASeq not available	Not Tested
SCC6T	WES	19	5455863	G	A	ZNRF4	p.G121S	Missense_Mutation	0.23958333	0	No	passenger	No	1	Clonal	RNASeq not available	Not Tested
SCC7T	WES	19	39191673	G	C	ACTN4	p.K103N	Missense_Mutation	0.19047619	0	No	likely_pathogenic	No	0.82	Clonal	RNASeq not available	Not Tested
SCC7T	WES	20	3804709	C	T	AP5S1	p.A123V	Missense_Mutation	0.19237435	0.02631579	No	passenger	No	0.82	Subclonal	RNASeq not available	Not Tested
SCC7T	WES	19	14000936	C	A	C19orf57	p.E245*	Nonsense_Mutation	0.18091451	0.01098901	No	passenger	No	0.78	Subclonal	RNASeq not available	Validated
SCC7T	WES	4	71201504	G	A	CABS1	p.D250N	Missense_Mutation	0.28048781	0.00383142	No	passenger	No	0.92	Clonal	RNASeq not available	Not Tested
SCC7T	WES	19	13325057	C	T	CACNA1A	p.R1977H	Missense_Mutation	0.29457364	0.03571429	No	passenger	No	1	Clonal	RNASeq not available	Validated
SCC7T	WES	16	83378554	C	T	CDH13	p.R289*	Nonsense_Mutation	0.07692308	0	No	passenger	No	0.25	Subclonal	RNASeq not available	Not Tested
SCC7T	WES	3	101477051	C	T	CEP97	p.S534L	Missense_Mutation	0.30693069	0.0167364	No	passenger	No	1	Clonal	RNASeq not available	Not Tested
SCC7T	WES	3	101477146	C	G	CEP97	p.Q566E	Missense_Mutation	0.36448598	0.00344828	No	passenger	No	1	Clonal	RNASeq not available	Not Validated
SCC7T	WES	3	101476002	C	G	CEP97	p.S330*	Nonsense_Mutation	0.35802469	0.01639344	No	passenger	No	1	Clonal	RNASeq not available	Validated
SCC7T	WES	3	101477188	C	T	CEP97	p.Q580*	Nonsense_Mutation	0.33103448	0.01602564	No	passenger	No	1	Clonal	RNASeq not available	Validated
SCC7T	WES	16	75513335	G	A	CHST6	p.S131L	Missense_Mutation	0.3625	0.0125523	No	likely_pathogenic	No	1	Clonal	RNASeq not available	Not Tested
SCC7T	WES	7	100875901	C	T	CLDN15	p.E189K	Missense_Mutation	0.21782178	0.01176471	No	passenger	No	0.72	Subclonal	RNASeq not available	Not Tested
SCC7T	WES	2	165578628	G	T	COBLL1	p.A318D	Missense_Mutation	0.04794521	0	No	passenger	No	0.16	Subclonal	RNASeq not available	Not Tested
SCC7T	WES	6	37012768	G	A	COX6A1P2	p.V45I	Missense_Mutation	0.1954023	0.02293578	No	passenger	No	0.84	Clonal	RNASeq not available	Not Tested
SCC7T	WES	4	169292904	C	T	DDX60L	p.R1596H	Missense_Mutation	0.35087719	0	No	passenger	No	1	Clonal	RNASeq not available	Not Tested
SCC7T	WES	10	127529516	C	G	DHX32	p.L531F	Missense_Mutation	0.34210526	0	No	passenger	No	1	Clonal	RNASeq not available	Not Tested
SCC7T	WES	6	7584499	C	T	DSP	p.A2335V	Missense_Mutation	0.07453416	0	No	passenger	No	0.25	Subclonal	RNASeq not available	Not Tested
SCC7T	WES	4	1801512	G	A	FGFR3	p.E140K	Missense_Mutation	0.27044025	0.03921569	No	passenger	No	0.89	Clonal	RNASeq not available	Validated
SCC7T	WES	3	30769850	G	C	GADL1	p.Q484E	Missense_Mutation	0.28571429	0.03149606	No	passenger	No	0.94	Clonal	RNASeq not available	Not Tested
SCC7T	WES	3	81692051	A	T	GBE1	p.H291Q	Missense_Mutation	0.35	0.0106383	No	likely_pathogenic	No	1	Clonal	RNASeq not available	Validated
SCC7T	WES	19	1083194	G	A	HMHA1	p.V949M	Missense_Mutation	0.2556391	0.03529412	No	passenger	No	1	Clonal	RNASeq not available	Not Tested
SCC7T	WES	19	10404743	G	C	ICAM5	p.C580S	Missense_Mutation	0.19832402	0.02164502	No	passenger	No	0.85	Clonal	RNASeq not available	Not Tested
SCC7T	WES	19	10407155	G	A	ICAM5	p.E880K	Missense_Mutation	0.16438356	0	No	passenger	No	0.7	Subclonal	RNASeq not available	Not Tested
SCC7T	WES	1	117122269	GGTC	G	IGSF3	p.D1046del	In_Frame_Del	0.21525886	0.00263158	Yes		No	0.71	Subclonal	RNASeq not available	Not Tested
SCC7T	WES	1	207074845	G	A	IL24	p.E105K	Missense_Mutation	0.25842697	0.00819672	Yes	passenger	No	0.85	Clonal	RNASeq not available	Validated
SCC7T	WES	2	234104059	G	C	INPP5D	p.E859Q	Missense_Mutation	0.29885058	0.00970874	No	likely_pathogenic	No	0.98	Clonal	RNASeq not available	Validated
SCC7T	WES	7	2645587	C	G	IQCE	p.I607M	Missense_Mutation	0.20289855	0.02255639	No	passenger	No	0.67	Subclonal	RNASeq not available	Not Tested
SCC7T	WES	1	62728920	G	T	KANK4	p.P795T	Missense_Mutation	0.22151899	0.03846154	Yes	passenger	No	0.73	Subclonal	RNASeq not available	Validated
SCC7T	WES	6	24576707	C	A	KIAA0319	p.L541F	Missense_Mutation	0.08415842	0	No	passenger	No	0.28	Subclonal	RNASeq not available	Not Tested
SCC7T	WES	23	118223123	C	A	KIAA1210	p.E690D	Missense_Mutation	0.2327044	0.012	No	passenger	No	0.76	Subclonal	RNASeq not available	Not Tested
SCC7T	WES	18	13884938	C	T	MC2R	p.V194M	Missense_Mutation	0.22828784	0.01529052	No	passenger	No	0.98	Clonal	RNASeq not available	Not Tested
SCC7T	WES	1	85412722	C	T	MCOLN2	p.G281R	Missense_Mutation	0.11764706	0	Yes	likely_pathogenic	No	0.39	Subclonal	RNASeq not available	Not Tested
SCC7T	WES	10	89312189	A	T	MINPP1	p.E473V	Missense_Mutation	0.28671329	0.01923077	No	passenger	No	0.94	Clonal	RNASeq not available	Not Tested
SCC7T	WES	15	23811156	C	A	MKRN3	p.P76Q	Missense_Mutation	0.24161074	0.02307692	No	passenger	No	0.79	Subclonal	RNASeq not available	Not Tested
SCC7T	WES	9	13106972	G	T	MPDZ	p.L2040I	Missense_Mutation	0.16666667	0	No	likely_pathogenic	No	0.38	Subclonal	RNASeq not available	Not Tested
SCC7T	WES	11	7064772	C	A	NLRP14	p.C505*	Nonsense_Mutation	0.28	0.00746269	No	passenger	No	0.92	Clonal	RNASeq not available	Validated
SCC7T	WES	12	80704484	A	G	OTOGL	p.I1134V	Missense_Mutation	0.05504587	0	No	passenger	No	0.18	Subclonal	RNASeq not available	Not Tested
SCC7T	WES	12	121600313	G	C	P2RX7	p.E175Q	Missense_Mutation	0.36094675	0.01714286	No	passenger	No	1	Clonal	RNASeq not available	Validated
SCC7T	WES	16	72156883	C	T	PMFBP1	p.A900T	Missense_Mutation	0.35820896	0.03278689	No	passenger	No	1	Clonal	RNASeq not available	Not Tested
SCC7T	WES	4	87695640	G	C	PTPN13	p.A1827P	Missense_Mutation	0.28813559	0	No	passenger	No	0.95	Clonal	RNASeq not available	Not Tested
SCC7T	WES	13	49096050	G	A	RCBTB2	p.S9F	Missense_Mutation	0.16666667	0	No	passenger	No	0.71	Subclonal	RNASeq not available	Not Tested
SCC7T	WES	3	196215543	G	A	RNF168	p.Q105*	Nonsense_Mutation	0.21568628	0.00680272	No	passenger	No	0.71	Subclonal	RNASeq not available	Not Validated
SCC7T	WES	20	1532380	G	C	SIRPD	p.I126M	Missense_Mutation	0.2173913	0.016	No	passenger	No	0.93	Clonal	RNASeq not available	Not Tested
SCC7T	WES	5	1057647	C	G	SLC12A7	p.E989Q	Missense_Mutation	0.30769231	0.01666667	No	likely_pathogenic	No	1	Clonal	RNASeq not available	Validated
SCC7T	WES	6	25915984	C	T	SLC17A2	p.R348Q	Missense_Mutation	0.05217391	0	No	passenger	No	0.17	Subclonal	RNASeq not available	Not Tested
SCC7T	WES	2	190428321	G	A	SLC40A1	p.A464V	Missense_Mutation	0.04807692	0	No	passenger	No	0.16	Subclonal	RNASeq not available	Not Tested
SCC7T	WES	23	70148046	C	T	SLC7A3	p.A257T	Missense_Mutation	0.28181818	0	No	likely_pathogenic	No	0.93	Clonal	RNASeq not available	Not Tested
SCC7T	WES	2	241987827	G	A	SNED1	p.E457K	Missense_Mutation	0.26356589	0.01851852	No	passenger	No	0.87	Clonal	RNASeq not available	Not Tested
SCC7T	WES	2	228882258	G	T	SPHKAP	p.S1104R	Missense_Mutation	0.33211679	0.02572347	No	passenger	No	1	Clonal	RNASeq not available	Not Tested
SCC7T	WES	13	33679753	C	T	STARD13	p.E1107K	Missense_Mutation	0.2335958	0.01416431	No	passenger	No	1	Clonal	RNASeq not available	Not Tested
SCC7T	WES	19	54577397	G	A	TARM1	p.Q145*	Nonsense_Mutation	0.1509434	0	No	passenger	No	0.65	Subclonal	RNASeq not available	Not Tested
SCC7T	WES	11	60694805	G	T	TMEM132A	p.R77L	Missense_Mutation	0.26128266	0.01655629	No	passenger	No	1	Clonal	RNASeq not available	Not Tested
SCC7T	WES	1	15546114	C	T	TMEM51	p.L213F	Missense_Mutation	0.28712871	0.01077586	Yes	passenger	No	0.94	Clonal	RNASeq not available	Validated
SCC7T	WES	11	63992057	G	A	TRPT1	p.H156Y	Missense_Mutation	0.17108434	0.02325581	No	passenger	No	0.73	Subclonal	RNASeq not available	Not Tested
SCC7T	WES	2	54483230	G	A	TSPYL6	p.P20L	Missense_Mutation	0.29032258	0.0125	No	passenger	No	0.95	Clonal	RNASeq not available	Not Tested
SCC7T	WES	2	74720257	G	A	TTC31	p.R491K	Missense_Mutation	0.33892617	0.02735562	No	passenger	No	1	Clonal	RNASeq not available	Not Tested
SCC7T	WES	21	43539414	G	A	UMODL1	p.R1018K	Missense_Mutation	0.23076923	0.02884615	No	passenger	No	0.99	Clonal	RNASeq not available	Not Tested
SCC7T	WES	16	11859390	G	C	ZC3H7A	p.F558L	Missense_Mutation	0.26857143	0.01895735	No	passenger	No	0.88	Clonal	RNASeq not available	Not Tested
SCC7T	WES	16	67429405	G	A	ZDHHC1	p.P331L	Missense_Mutation	0.32835821	0.02112676	No	passenger	No	1	Clonal	RNASeq not available	Not Tested
SCC3T	MSK-IMPACT	23	63411972	C	A	AMER1	p.E399*	Nonsense_Mutation	0.30924856	0	No	likely_pathogenic	No	0.93	Clonal	RNASeq not available	Not Tested
SCC3T	MSK-IMPACT	23	76938197	C	T	ATRX	p.E851K	Missense_Mutation	0.18518519	0	No	likely_pathogenic	No	0.55	Subclonal	RNASeq not available	Not Tested
SCC3T	MSK-IMPACT	2	25463182	G	A	DNMT3A	p.R771*	Nonsense_Mutation	0.34883721	0.00423729	No	likely_pathogenic	No	0.7	Subclonal	RNASeq not available	Not Tested
SCC3T	MSK-IMPACT	7	6018323	G	A	PMS2	p.Q727*	Nonsense_Mutation	0.18213058	0	Yes	likely_pathogenic	No	0.36	Subclonal	RNASeq not available	Not Tested
SCC3T	MSK-IMPACT	23	47045891	G	A	RBM10	p.G896S	Missense_Mutation	0.15631692	0	No	passenger	No	0.47	Subclonal	RNASeq not available	Not Tested
SCC3T	MSK-IMPACT	4	69512845	C	T	UGT2B15	p.G524R	Missense_Mutation	0.04060914	0.00606061	Yes	passenger	No	0.08	Subclonal	RNASeq not available	Not Tested
SCC9T	MSK-IMPACT	2	212288922	G	C	ERBB4	p.P942A	Missense_Mutation	0.40493827	0	Yes	likely_pathogenic	No	1	Clonal	RNASeq not available	Not Tested
SCC9T	MSK-IMPACT	17	29585360	A	T	NF1	p.X1392_splice	Splice_Site	0.37539432	0	No	likely_pathogenic	No	1	Clonal	RNASeq not available	Not Tested
SCC9T	MSK-IMPACT	17	29528454	C	T	NF1	p.S404F	Missense_Mutation	0.34864865	0	No	passenger	No	0.94	Clonal	RNASeq not available	Not Tested
SCC9T	MSK-IMPACT	17	29527584	C	T	NF1	p.L345F	Missense_Mutation	0.30508475	0	No	passenger	No	0.83	Subclonal	RNASeq not available	Not Tested
SCC9T	MSK-IMPACT	17	29527590	C	T	NF1	p.Q347*	Nonsense_Mutation	0.30449827	0	No	likely_pathogenic	No	0.82	Subclonal	RNASeq not available	Not Tested
SCC9T	MSK-IMPACT	19	5221011	T	C	PTPRS	p.Q1152R	Missense_Mutation	0.0531401	0	No	passenger	No	0.2	Subclonal	RNASeq not available	Not Tested
SCC9T	MSK-IMPACT	1	17349165	CAGCTTGG	G	SDHB	p.A232_L234de	In_Frame_Del	0.26068376	0	No	potentially_pathogenic	No	0.71	Subclonal	RNASeq not available	Not Tested
SCC9T	MSK-IMPACT	17	7577538	C	T	TP53	p.R248Q	Missense_Mutation	0.35341366	0	No	likely_pathogenic	Yes	0.96	Clonal	RNASeq not available	Not Tested

Supplementary Table S5. Putative in-frame fusion transcripts with driver probability >0.1 identified in SCC1 and SCC2 using RNA-sequencing.

Sample ID	Genes	Genomic coordinates	Spanning and split reads (n)	Split reads (n)	Driver probability (Oncofuse)	Algorithm	5' location	3' location	P-value
SCC2T	VMP1-RPS6KB1	chr17:57842500>chr17:57987922	3	3	0.997438	chimerascan	Intron	Intron	0.04913752
SCC2T	SLC35G2-NCK1	chr3:136538057>chr3:136646825	2	1	0.9961532	chimerascan	Exon	Intron	0.05754801
SCC1T	DMC1-DDX17	chr22:38917612>chr22:38897286	3	2	0.9858879	chimerascan	Intron	Intron	0.14232601
SCC1T	ABCA10-ABCA6	chr17:67145173>chr17:67136890	2	2	0.9736921	chimerascan	Intron	Intron	0.19960184
SCC1T	ARFIP2-TRIM3	chr11:6498947>chr11:6486963	2	2	0.9446882	chimerascan	Intron	Intron	0.34136487
SCC2T	PDLIM2-C8orf58	chr8:22447255>chr8:22458394	2	2	0.8289519	chimerascan	Intron	Intron	0.63653723
SCC2T	GKAP1-KIF27	chr9:86431910>chr9:86530594	7	2	0.5981402	chimerascan	Intron	Intron	1
SCC2T	GKAP1-KIF27	chr9:86431910>chr9:86530594	28	17	0.5981402	defuse	Intron	Intron	1
SCC1T	POLA2-CDC42EP2	chr11:65063462>chr11:65088014	4	3	0.5912114	chimerascan	Intron	Intron	1
SCC2T	POLA2-CDC42EP2	chr11:65063462>chr11:65088014	23	10	0.5912114	chimerascan	Intron	Intron	1
SCC2T	POLA2-CDC42EP2	chr11:65063462>chr11:65088014	41	21	0.5912114	defuse	Intron	Intron	0.97330609
SCC2T	PIBF1-KLF5	chr13:73573134>chr13:73635998	2	2	0.5481406	chimerascan	Intron	Intron	1
SCC1T	TLK2-FAM157A	chr17:60637488>chr3:197907625	33	33	0.2722771	chimerascan	Intron	Intron	1
SCC2T	TLK2-FAM157A	chr17:60637488>chr3:197907625	44	29	0.2722771	chimerascan	Intron	Intron	1
SCC1T	TLK2-FAM157B	chr17:60637488>chr9:141134069	33	33	0.2722771	chimerascan	Intron	Intron	1
SCC2T	HOXB6-HOXB3	chr17:46681841>chr17:46651377	3	2	0.1747878	chimerascan	Intron	Exon	1
SCC1T	ZNF716-ZNF678	chr7:57522293>chr1:227842036	4	4	0.1540229	chimerascan	Intron	Intron	1
SCC1T	F8-TMLHE	chrX:154114408>chrX:154718952	2	1	0.1096824	chimerascan	Intron	Exon	1
SCC1T	MBD1-CCDC11	chr18:47799193>chr18:47788590	3	2	0.1056514	chimerascan	Intron	Intron	1

Supplementary Table S6. Putative HPV integration sites based on RNA-sequencing.

Sample ID	hg19 locus	hg19 annotation	Mate 1 HPV18 Position	Mate 2 hg19 Chromosome 18 Position
SCC1T	18q12.3	-	882	39411292
SCC1T	18q12.3	-	2280	39497187
SCC1T	18q12.3	-	3669	39401088
SCC2T	8p22	RP11-89M16.1-002 (lncRNA)	2603	129517175
SCC2T	8p22	RP11-89M16.1-002 (lncRNA)	4908	129518183
SCC2T	8p22	RP11-89M16.1-002 (lncRNA)	5996	129517009
SCC2T	8p22	RP11-89M16.1-002 (lncRNA)	5998	129509523
